# Physical layer covert communication for UAV-aided transportation systems via rate-splitting multiple access over Nakagami-*m* fading

**DOI:** 10.1371/journal.pone.0361873

**Published:** 2026-11-12

**Authors:** Nghia Hoang Trong, Anh Le-Thi, Trung-Anh Do, Hoa Tien Nguyen, Quang-Sang Nguyen

**Affiliations:** 1 Faculty of Electrical and Electronic Engineering, Hanoi Open University, Hanoi, Vietnam; 2 School of Information and Communications Technology, Hanoi University of Industry, Hanoi, Vietnam; 3 Posts and Telecommunications Institute of Technology, Hanoi, Vietnam; 4 School of Electrical and Electronic Engineering, Hanoi University of Science and Technology, Hanoi, Vietnam; 5 Posts and Telecommunications Institute of Technology, Ho Chi Minh City, Vietnam; Sultan Qaboos University, OMAN

## Abstract

Unmanned aerial vehicles (UAVs) have recently emerged as a promising solution for supporting intelligent transportation systems by providing flexible and reliable wireless connectivity among vehicular users. However, the broadcast nature of wireless channels exposes vehicular communications to monitoring and interception by unauthorized vehicles. In this paper, we investigate physical-layer covert communication in a UAV-assisted vehicular network employing rate-splitting multiple access (RSMA) over Nakagami-*m* fading channels. In the considered system, the UAV simultaneously transmits common and private messages to multiple vehicles, while an eavesdropper vehicle $V_{E}$ attempts to monitor and intercept the transmission. To enhance communication covertness, a friendly vehicular jammer with randomly distributed transmit power is introduced to increase uncertainty at the monitoring vehicle. Closed-form expressions for the outage probabilities of legitimate vehicles are derived to evaluate communication reliability, while the detection error probability and intercept probability are analyzed to characterize covert and security performance. In addition, asymptotic outage analysis is conducted to reveal the diversity behavior determined by Nakagami fading parameters. Monte Carlo simulations verify the accuracy of the analytical results and illustrate the impacts of key system parameters, including power allocation, fading severity, and random jamming power. The results provide useful design insights for secure UAV-assisted transportation communication systems.

## 1 Introduction

The rapid evolution of intelligent transportation systems (ITSs) has driven the demand for highly reliable, secure, and flexible wireless communication solutions. The integration of UAVs into transportation networks has recently emerged as a promising paradigm due to their dynamic deployment capability, wide coverage, and strong line-of-sight transmission characteristics. UAV-assisted communication platforms are particularly suitable for supporting data exchange among autonomous vehicles, traffic monitoring infrastructure, and edge computing nodes, especially in complex urban environments where terrestrial networks may experience coverage limitations [1].

Despite these advantages, UAV-enabled vehicular communication systems remain vulnerable to security and privacy threats because of the broadcast nature of wireless channels. Traditional encryption-based security solutions may introduce significant computational overhead and latency, which are not always suitable for delay-sensitive transportation applications. Consequently, physical layer security techniques have gained increasing attention as lightweight and effective alternatives for protecting confidential vehicular information [2–4].

Recently, covert communication has been proposed as a promising approach that hides transmission activities from eavesdropper vehicles by ensuring that communication remains statistically indistinguishable from background noise [5,6]. Meanwhile, RSMA has been recognized as an efficient multiple access framework capable of improving spectral efficiency, user fairness, and interference management in multi-user wireless systems [7,8]. Motivated by these observations, this work investigates covert vehicular communication in UAV-assisted transportation networks using RSMA transmission over generalized Nakagami-*m* fading channels. In addition, friendly jamming with random transmit power is incorporated to further enhance covertness performance.

### 1.1 Related work

UAV-assisted wireless communication has attracted considerable research attention in recent years due to its ability to extend network coverage and improve transmission reliability. Several studies have investigated UAV-enabled communication systems under various fading conditions and system configurations. For instance, UAV-assisted transmission under generalized fading channels has been analyzed to evaluate outage performance and reliability characteristics [9–11]. Other works have explored UAV-based cooperative relaying and aerial base station deployment to enhance connectivity in vehicular and transportation networks [12–14]. However, most of these studies primarily focus on reliability performance and do not consider advanced multiple access techniques or covert communication requirements.

In parallel, RSMA has been widely investigated as a flexible multiple access technique that bridges the gap between orthogonal and non-orthogonal multiple access schemes. Existing works have demonstrated that RSMA can effectively manage multi-user interference and improve spectral efficiency in heterogeneous wireless networks [15–17]. Several studies have further extended RSMA frameworks to UAV-enabled networks and vehicular communication scenarios, showing significant improvements in transmission performance and user fairness [18–20]. Nevertheless, the application of RSMA in covert vehicular communication remains relatively limited.

Covert communication has recently emerged as a promising physical-layer security mechanism that ensures low probability of detection at unauthorized eavesdropper vehicles. Prior studies have analyzed covert communication performance under various wireless environments, including relay-assisted networks, cognitive radio systems, and UAV-enabled communication frameworks [21–23]. These works typically evaluate detection error probability and covert throughput under different channel and network configurations. Furthermore, friendly jamming has been shown to effectively enhance covert communication performance by intentionally generating interference to introduce additional uncertainty at eavesdropper receivers [24–30]. However, most existing studies consider fixed or deterministic jamming strategies and do not investigate random power jamming models in UAV-assisted transportation scenarios.

Although several recent works have attempted to integrate covert communication with RSMA transmission, these studies mainly focus on terrestrial communication systems or simplified network configurations [31–33]. The joint investigation of UAV-assisted vehicular communication, RSMA transmission, covert communication, and friendly jamming over generalized Nakagami-*m* fading channels remains largely unexplored. Moreover, comprehensive performance evaluations, including outage probability (OP), detection error probability (DEP), intercept probability (IP), and system throughput, have not been fully addressed in a unified analytical framework.

### 1.2 Motivation and contributions

Recent developments in UAV-assisted wireless transportation networks have attracted growing research interest, mainly because UAV platforms can provide flexible coverage and on-demand communication support for intelligent vehicular applications. Several studies have explored UAV-enabled transmission under different channel models and access strategies. For instance, UAV-assisted communication over Nakagami-*m* fading channels was investigated in [34], where the authors mainly focused on reliability analysis and asymptotic performance in the high-SNR region.

Along another direction, RSMA has recently been considered a promising multiple access framework due to its ability to balance interference management and spectral efficiency. The potential of RSMA in wireless systems has been examined in [35–37] under various communication scenarios. Meanwhile, covert communication has gradually emerged as a practical physical-layer security mechanism that attempts to conceal transmission activities from unauthorized monitoring vehicles.

Despite these encouraging results, some important issues remain insufficiently addressed. As summarized in Table 1, most existing studies typically focus on only one or two key technologies, while the combined utilization of UAV-assisted transmission, RSMA, and covert communication for transportation communication systems has not been fully investigated. More specifically, the work in [34] considers UAV-assisted communication over Nakagami-*m* fading channels but does not address covert transmission or RSMA-based signaling. The study in [35] investigates covert communication using RSMA; however, UAV-assisted vehicular communication scenarios and reliability metrics such as OP and high-SNR behavior are not examined. In [38], the authors analyze covert communication performance but do not incorporate RSMA transmission or friendly jamming techniques. Similarly, the works in [36] and [37] consider RSMA-based communication and covert transmission, yet friendly jamming strategies and IP analysis in UAV-assisted transportation environments are still missing.

**Table 1:** Comparisons of the proposed work with existing literature.

Context	[34]	[35]	[38]	[36]	[37]	Proposed
UAV	✓	✗	✗	✓	✓	✓
RSMA	✗	✓	✗	✓	✓	✓
Nakagami-*m* fading	✓	✗	✗	✓	✓	✓
Friendly Jamming	✗	✓	✗	✗	✗	✓
Covert Communication	✗	✓	✓	✗	✓	✓
OP	✓	✗	✓	✓	✓	✓
High SNR Region	✓	✗	✓	✓	✓	✓
Sum throughput	✗	✗	✗	✗	✗	✓
DEP	✗	✗	✓	✗	✓	✓
IP	✗	✗	✗	✗	✗	✓

In addition, friendly jamming has been widely recognized as an effective approach for improving covert communication performance by introducing additional uncertainty at monitoring nodes. However, most existing studies assume deterministic or fixed jamming power models, which may not accurately reflect realistic vehicular transportation environments where mobile jamming vehicles introduce dynamic and random interference conditions. Furthermore, IP and DEP have not been jointly analyzed in RSMA-based UAV transportation systems over generalized Nakagami-*m* fading channels. Moreover, performance metrics such as sum throughput have rarely been investigated in covert vehicular communication scenarios.

Motivated by the above observations, this paper proposes a physical-layer covert communication framework for UAV-assisted transportation systems employing RSMA over Nakagami-*m* fading channels with the assistance of a vehicle-based jammer having randomly distributed transmit power. The core novelty of this work lies in quantifying the uncertainty introduced by a friendly vehicular jammer with randomly distributed transmit power within an RSMA framework. Unlike prior works that assume fixed jamming power or simpler fading models, we provide exact closed-form analytical solutions over Nakagami-*m* fading channels. This derivation reveals genuinely novel design insights into the trade-off between power allocation and covertness in UAV-assisted ITS. The main contributions of this paper are summarized as follows:

- A novel UAV-assisted RSMA transportation communication model is developed, where covert transmission is performed in the presence of an eavesdropper vehicle $V_{E}$ and a friendly vehicular jammer with random transmit power, capturing realistic vehicular interference dynamics.
- Closed-form analytical expressions for the OPs of legitimate vehicles and the IPs at eavesdropper vehicle $V_{E}$ are derived to evaluate communication reliability and information security performance. Meanwhile, an analytical approximation for the DEP at monitoring vehicle $V_{E}$ is developed to quantify covertness performance.
- High-SNR asymptotic analyses are developed to provide diversity insights into the proposed system under different fading severity conditions.
- The impacts of key system parameters, including RSMA power allocation, Nakagami fading parameters, and random jamming power, are comprehensively investigated through both analytical and Monte Carlo simulation results, offering useful design guidelines for secure UAV-assisted transportation networks.

### 1.3 Paper organization and notations

The remainder of this paper is organized as follows. Section 2 presents the system model and formulates the covert vehicular communication framework. Section 3 derives closed-form analytical expressions for the OPs, IPs, and sum throughput, as well as an analytical approximation for the DEP, along with the corresponding asymptotic analyses. Section 4 presents numerical and simulation results to validate the analytical derivations and provide performance insights. Finally, Section 5 concludes the paper.

**Notations:** The cumulative distribution function (CDF) and probability density function (PDF) of *X* are represented as $F_{X}\left(.\right)$ and $f_{X}\left(.\right)$, respectively. The operators Pr(·) and 𝔼{·} denote probability and expectation, respectively. The Gamma function is denoted by Γ(·), and the lower and upper incomplete Gamma functions are represented by γ(·,·) and Γ(·,·), respectively. The notation |·| denotes the absolute value of a complex variable. The symbol ~ denotes statistical distribution and 𝒪(·) represents the asymptotic order.

## 2 System model

Fig 1 illustrates a UAV-enabled RSMA vehicular network, consisting of a UAV, a friendly jammer vehicle ($V_{J}$), two legitimate RSMA vehicles (the secure vehicle $V_{S}$ and interference vehicle $V_{I}$), and an eavesdropper vehicle ($V_{E}$) equipped with both monitoring and eavesdropping capabilities. For analytical convenience, the UAV is assumed to hover at a fixed altitude, which may reflect practical deployment scenarios where maintaining a stable aerial platform is preferred for reliable vehicular coverage. All nodes are equipped with a single antenna and operate in half-duplex mode.

**Fig 1:**
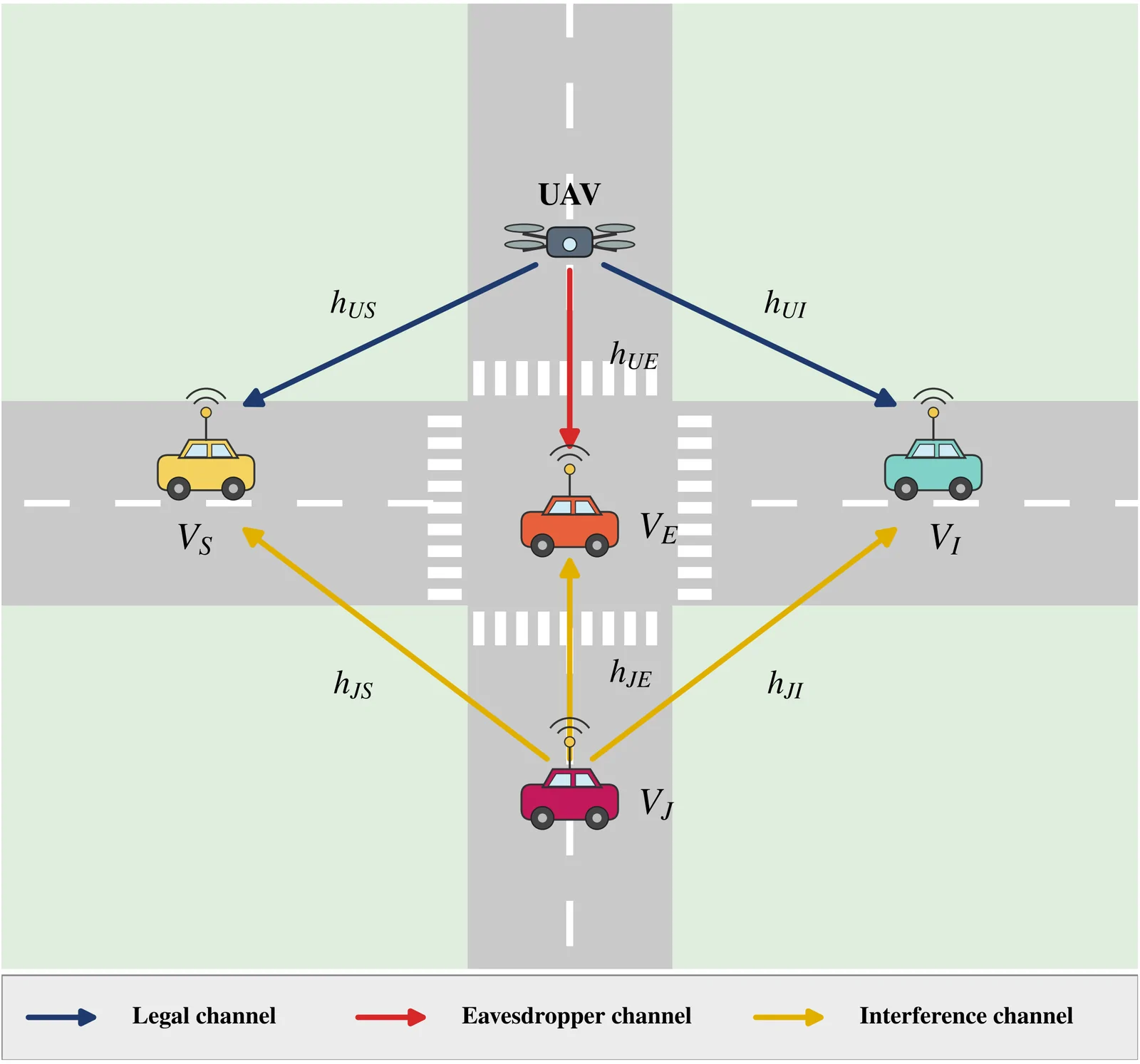
System model of the proposed UAV-enabled RSMA vehicular network with covert communication and jamming assistance.

We consider air-to-ground wireless links between the UAV and ground vehicles. The channel between the UAV and $V_{a}$, where a∈{I,S,E}, is modeled by incorporating both small-scale fading and large-scale path-loss effects. The overall channel gain is expressed as


$$\begin{array}{c} g_{Ua}=h_{Ua}\sqrt{\chi_{Ua}}, \end{array}$$ (1)

where $h_{Ua}$ represents the small-scale fading coefficient, while $\chi_{Ua}$ characterizes the large-scale fading component. The large-scale fading is modeled as


$$\begin{array}{c} \chi_{Ua}={\left(\frac{c}{4\pi f}\right)}^{2}d_{Ua}^{-\alpha }, \end{array}$$ (2)

where *c* denotes the speed of light, *f* is the carrier frequency, and α represents the path-loss exponent.

The distance between the UAV located at $\left(x_{U},y_{U},z_{U}\right)$ and the vehicle $V_{a}$ positioned at $\left(x_{a},y_{a},z_{a}\right)$ is given by


$$\begin{array}{c} d_{Ua}=\sqrt{(x_{U}-x_{a}{)}^{2}+(y_{U}-y_{a}{)}^{2}+(z_{U}-z_{a}{)}^{2}}. \end{array}$$ (3)

Under the protection of $V_{J}$, the UAV transmits superimposed RSMA signals to $V_{S}$ and $V_{I}$ in the presence of eavesdropper vehicle $V_{E}$. After message splitting and precoding, the transmitted signal $s_{b}$ can be expressed as


$$\begin{array}{c} s_{b}=\{\begin{array}{cc} {\bar{x}}_{c}\sqrt{P_{b}w_{c}}+{\bar{x}}_{S}\sqrt{P_{b}w_{S}}+{\bar{x}}_{I}\sqrt{P_{b}w_{I}}=s_{b1}, & H_{1},\\ {\bar{x}}_{c}\sqrt{P_{b}w_{c}}+{\bar{x}}_{I}\sqrt{P_{b}w_{I}}=s_{b0}, & H_{0}, \end{array} \end{array}$$ (4)

Here, $P_{b}$ denotes the total transmit power at the UAV. The transmitted symbols satisfy $𝔼\left\{{\left|{\bar{x}}_{q}\right|}^{2}\right\}=1$, where q∈{c,S,I}. The parameters $w_{c}$, $w_{S}$, and $w_{I}$ represent the power allocation coefficients assigned to the common message and the private messages of $V_{S}$ and $V_{I}$, respectively, which satisfy the constraint $w_{c}+w_{S}+w_{I}=1$.

It is worth noting that $V_{J}$ continuously transmits an artificial interference signal given by $s_{J}={\bar{x}}_{J}\sqrt{P_{J}}$, where $𝔼\left\{{\left|{\bar{x}}_{J}\right|}^{2}\right\}=1$. The jamming power $P_{J}$ is modeled as a random variable following a continuous uniform distribution over the interval $\left[0,{\bar{P}}_{J}\right]$, i.e., $P_{J}\sim 𝒰\left(0,{\bar{P}}_{J}\right)$, whose PDF is given by


$$\begin{array}{c} f_{P_{J}}(x)=\left\{ \begin{array}{cc} {\bar{P}}_{J}^{-1}, & 0\leq x\leq {\bar{P}}_{J},\\ 0, & \text{otherwise}. \end{array}\right. \end{array}$$ (5)

Nakagami-*m* fading is adopted to model the small-scale fading characteristics of all wireless links. The PDFs of the random variables (RVs) ${\left|h_{Ua}\right|}^{2}$ and ${\left|h_{Ja}\right|}^{2}$ are given by [39, Eq. (6)]


$$\begin{array}{c} f_{{\left|h_{Ua}\right|}^{2}}(x)=\frac{\mu_{Ua}^{m_{Ua}}x^{m_{Ua}-1}}{\Gamma (m_{Ua})}e^{-\mu_{Ua}x}, \end{array}$$ (6a)

$$\begin{array}{c} f_{{\left|h_{Ja}\right|}^{2}}(x)=\frac{\mu_{Ja}^{m_{Ja}}x^{m_{Ja}-1}}{\Gamma (m_{Ja})}e^{-\mu_{Ja}x}, \end{array}$$ (6b)

where Γ(·) denotes the Gamma function [40, Eq. (8.310)], and $\mu_{Ua}=\frac{m_{Ua}}{\lambda_{Ua}}$ and $\mu_{Ja}=\frac{m_{Ja}}{\lambda_{Ja}}$ represent the fading parameters of the UAV and jammer links, respectively. Here, $m_{Ua}$ and $m_{Ja}$ denote the shape parameters, while $\lambda_{Ua}$ and $\lambda_{Ja}$ represent the spread parameters of the Nakagami-*m* distribution.

Based on [41], the CDFs of the instantaneous channel gains ${\left|h_{Ua}\right|}^{2}$ and ${\left|h_{Ja}\right|}^{2}$ can be expressed as


$$\begin{array}{c} F_{{\left|h_{Ua}\right|}^{2}}(x)=\frac{\gamma (m_{Ua},\mu_{Ua}x)}{\Gamma (m_{Ua})}=1-e^{-\mu_{Ua}x}\sum_{t=0}^{m_{Ua}-1}\frac{(\mu_{Ua}x{)}^{t}}{t!}, \end{array}$$ (7a)

$$\begin{array}{c} F_{{\left|h_{Ja}\right|}^{2}}(x)=\frac{\gamma (m_{Ja},\mu_{Ja}x)}{\Gamma (m_{Ja})}=1-e^{-\mu_{Ja}x}\sum_{t=0}^{m_{Ja}-1}\frac{(\mu_{Ja}x{)}^{t}}{t!}, \end{array}$$ (7b)

where γ(·,·) denotes the lower incomplete Gamma function [40, Eq. (8.350.1)].

The vehicles $V_{S}$, $V_{I}$, and $V_{E}$ receive the transmitted signal $s_{b}$ from the direct UAV link. The corresponding received signals are expressed as


$$\begin{array}{cc} {\bar{y}}_{US}= & g_{US}s_{b}+\sqrt{d_{JS}^{-\alpha }P_{J}}h_{JS}s_{J}+\omega_{US}\\ = & h_{US}\sqrt{\chi_{US}}s_{b}+\sqrt{d_{JS}^{-\alpha }P_{J}}h_{JS}s_{J}+\omega_{US}, \end{array}$$ (8a)

$$\begin{array}{cc} {\bar{y}}_{UI}= & g_{UI}s_{b}+\sqrt{d_{JI}^{-\alpha }P_{J}}h_{JI}s_{J}+\omega_{UI}\\ = & h_{UI}\sqrt{\chi_{UI}}s_{b}+\sqrt{d_{JI}^{-\alpha }P_{J}}h_{JI}s_{J}+\omega_{UI}, \end{array}$$ (8b)

$$\begin{array}{cc} {\bar{y}}_{UE}= & g_{UE}s_{b}+\sqrt{d_{JE}^{-\alpha }P_{J}}h_{JE}s_{J}+\omega_{UE}\\ = & h_{UE}\sqrt{\chi_{UE}}s_{b}+\sqrt{d_{JE}^{-\alpha }P_{J}}h_{JE}s_{J}+\omega_{UE}, \end{array}$$ (8c)

where $d_{JS}$, $d_{JI}$, and $d_{JE}$ denote the distances between the jammer vehicle $V_{J}$ and $V_{S}$, $V_{I}$, and $V_{E}$, respectively. For analytical tractability, the friendly jammer is assumed to be located at a fixed ground position. Therefore, the distances between $V_{J}$ and each vehicle are modeled as deterministic constants. The terms $\omega_{US}$, $\omega_{UI}$, and $\omega_{UE}$ denote additive white Gaussian noise (AWGN) with zero mean and variance $\sigma_{S}^{2}$, $\sigma_{I}^{2}$, and $\sigma_{E}^{2}$ at $V_{S}$, $V_{I}$, and $V_{E}$, respectively.

The information intended for $V_{S}$ and $V_{I}$ consists of two components, namely the common message (${\bar{x}}_{c}$) and the private messages (${\bar{x}}_{S}$ or ${\bar{x}}_{I}$). Following the RSMA decoding procedure, both vehicles first decode the common message while treating the remaining signals as interference. After successfully removing the common message using successive interference cancellation (SIC), each vehicle proceeds to decode its corresponding private message.

### 2.1 SINR computation for $V_{S}$

When $V_{S}$ decodes the common message ${\bar{x}}_{c}$, the signal-to-interference-plus-noise ratio (SINR) is expressed as


$$\begin{array}{c} {\bar{\gamma }}_{S}^{{\bar{x}}_{c}}=\frac{w_{c}\chi_{US}P_{b}{\left|h_{US}\right|}^{2}}{\left(w_{S}+w_{I}\right)\chi_{US}P_{b}{\left|h_{US}\right|}^{2}+\eta d_{JS}^{-\alpha }P_{J}{\left|h_{JS}\right|}^{2}+\sigma_{S}^{2}}. \end{array}$$ (9)

After successfully decoding and removing the common message using successive interference cancellation (SIC), the SINR for decoding the private message ${\bar{x}}_{S}$ can be written as


$$\begin{array}{c} {\bar{\gamma }}_{S}^{{\bar{x}}_{S}}=\frac{w_{S}\chi_{US}P_{b}{\left|h_{US}\right|}^{2}}{w_{I}\chi_{US}P_{b}{\left|h_{US}\right|}^{2}+\eta d_{JS}^{-\alpha }P_{J}{\left|h_{JS}\right|}^{2}+\sigma_{S}^{2}}, \end{array}$$ (10)

where η∈[0,1] denotes the residual level of interference, in which η=0 and η=1 indicate that the interference is eliminated and unmitigated, respectively. In this work, it is assumed that the jamming signal generated by $V_{J}$ cannot be completely eliminated.

**Remark 1.** *If the covert information* ${\bar{x}}_{S}$ *is transmitted (i.e.,* $s_{b}=s_{b1}$*), the decoding SINR at* $V_{S}$ *depends on both (9) and (10). Otherwise, when* $s_{b}=s_{b0}$*, only (9) is required for decoding.*

### 2.2 SINR computation for $V_{I}$

Vehicle $V_{I}$ decodes both the common message and its corresponding private message regardless of whether the transmitted signal contains the covert message ${\bar{x}}_{S}$. Therefore, the SINR expressions can be unified as follows.

When decoding the common message ${\bar{x}}_{c}$, the SINR at $V_{I}$ is given by


$$\begin{array}{c} {\bar{\gamma }}_{I}^{{\bar{x}}_{c}}=\frac{w_{c}\chi_{UI}P_{b}{\left|h_{UI}\right|}^{2}}{\left(w_{S}+w_{I}\right)\chi_{UI}P_{b}{\left|h_{UI}\right|}^{2}+\eta d_{JI}^{-\alpha }P_{J}{\left|h_{JI}\right|}^{2}+\sigma_{I}^{2}}. \end{array}$$ (11)

After removing the common message via SIC, the SINR for decoding the private message ${\bar{x}}_{I}$ can be expressed as


$$\begin{array}{c} {\bar{\gamma }}_{I}^{{\bar{x}}_{I}}=\frac{w_{I}\chi_{UI}P_{b}{\left|h_{UI}\right|}^{2}}{w_{S}\chi_{UI}P_{b}{\left|h_{UI}\right|}^{2}+\eta d_{JI}^{-\alpha }P_{J}{\left|h_{JI}\right|}^{2}+\sigma_{I}^{2}}. \end{array}$$ (12)

where η∈[0,1] is the residual level of interference.

### 2.3 Signal monitoring and interception strategy at $V_{E}$

In this paper, $V_{E}$ is assumed to possess both monitoring and interception capabilities. To efficiently eavesdrop on the covert message ${\bar{x}}_{S}$ while conserving computational and energy resources, $V_{E}$ operates according to the following procedure:

1. $V_{E}$ first attempts to decode the common message ${\bar{x}}_{c}$ by treating all remaining signals as interference, regardless of whether the transmitted signal $s_{b}$ contains ${\bar{x}}_{S}$.
2. After removing the common message, the remaining received signal power, denoted by ${\hat{P}}_{E}$, is divided into two portions using a power allocation factor ℓ∈[0,1]. In particular, $V_{E}$ utilizes $\ell {\hat{P}}_{E}$ to monitor whether the residual received signal contains the covert message ${\bar{x}}_{S}$.
3. If the presence of ${\bar{x}}_{S}$ is confirmed, $V_{E}$ employs the remaining power $(1-\ell ){\hat{P}}_{E}$ to decode ${\bar{x}}_{S}$. Otherwise, $V_{E}$ terminates the decoding process to avoid unnecessary resource consumption.

#### 2.3.1 SINR for decoding ${\bar{x}}_{c}$.

Following the operational mechanism of $V_{E}$, the SINR for decoding the common message ${\bar{x}}_{c}$ is expressed as


$$\begin{array}{c} {\bar{\gamma }}_{E}^{{\bar{x}}_{c}}=\frac{w_{c}\chi_{UE}P_{b}{\left|h_{UE}\right|}^{2}}{\left(w_{S}+w_{I}\right)\chi_{UE}P_{b}{\left|h_{UE}\right|}^{2}+d_{JE}^{-\alpha }P_{J}{\left|h_{JE}\right|}^{2}+\sigma_{E}^{2}}. \end{array}$$ (13)

#### 2.3.2 Average monitoring power for detecting ${\bar{x}}_{S}$.

Based on the received signal and the Neyman–Pearson detection framework, $V_{E}$ faces a binary hypothesis testing problem:

- $H_{0}$: The UAV does not transmit the covert signal ${\bar{x}}_{S}$.
- $H_{1}$: The UAV transmits the covert signal ${\bar{x}}_{S}$.

Accordingly, the received signal at $V_{E}$ under the two hypotheses can be written as


$$\begin{array}{c} {\hat{y}}_{E}=\left\{ \begin{array}{c} H_{0}:h_{UE}\sqrt{\chi_{UE}}s_{b0}+\sqrt{d_{JE}^{-\alpha }P_{J}}h_{JE}s_{J}+\omega_{UE},\\ H_{1}:h_{UE}\sqrt{\chi_{UE}}s_{b1}+\sqrt{d_{JE}^{-\alpha }P_{J}}h_{JE}s_{J}+\omega_{UE}. \end{array}\right. \end{array}$$ (14)

Following [38], the average received signal power is defined as


$$\begin{array}{c} {\hat{P}}_{E}=\frac{1}{P}\sum_{p=1}^{P}{\left|{\hat{y}}_{E}(p)\right|}^{2}. \end{array}$$ (15)

Assuming an infinite number of signal samples, i.e., P→∞, the average received power after removing the common message ${\bar{x}}_{c}$ is obtained as


$$\begin{array}{c} {\hat{P}}_{E}=\left\{ \begin{array}{cc} \varsigma_{0}P_{b}{\left|h_{UE}\right|}^{2}+d_{JE}^{-\alpha }P_{J}{\left|h_{JE}\right|}^{2}+\sigma_{E}^{2}, & H_{0},\\ \varsigma_{1}P_{b}{\left|h_{UE}\right|}^{2}+d_{JE}^{-\alpha }P_{J}{\left|h_{JE}\right|}^{2}+\sigma_{E}^{2}, & H_{1}, \end{array}\right. \end{array}$$ (16)

where $\varsigma_{1}=\left(\eta w_{c}+w_{I}+w_{S}\right)\chi_{UE}$ and $\varsigma_{0}=\left(\eta w_{c}+w_{I}\right)\chi_{UE}$. Here, η∈[0,1] represents the residual degree of imperfect cancellation for the common message ${\bar{x}}_{c}$ at $V_{E}$.

Since only a portion of the available power is used for monitoring, the monitoring test statistic is expressed as


$$\begin{array}{c} T_{E}=\left\{ \begin{array}{cc} \ell \left(\varsigma_{0}P_{b}{\left|h_{UE}\right|}^{2}+d_{JE}^{-\alpha }P_{J}{\left|h_{JE}\right|}^{2}+\sigma_{E}^{2}\right), & H_{0},\\ \ell \left(\varsigma_{1}P_{b}{\left|h_{UE}\right|}^{2}+d_{JE}^{-\alpha }P_{J}{\left|h_{JE}\right|}^{2}+\sigma_{E}^{2}\right), & H_{1}. \end{array}\right. \end{array}$$ (17)

The decision rule is given by


$$\begin{array}{c} T_{E}\overset{D_{1}}{\underset{D_{0}}{>rless}}\tau , \end{array}$$ (18)

where τ denotes the detection threshold. If $T_{E}>\tau$, $V_{E}$ decides that the UAV transmits ${\bar{x}}_{S}$; otherwise, $V_{E}$ decides that no covert transmission occurs.

#### 2.3.3 SINR for decoding ${\bar{x}}_{S}$.

To simplify the analysis, we consider the worst-case scenario where $V_{E}$ successfully detects the existence of ${\bar{x}}_{S}$. The remaining power used for decoding ${\bar{x}}_{S}$ is given by


$$\begin{array}{c} (1-\ell )\left(\varsigma_{1}P_{b}{\left|h_{UE}\right|}^{2}+d_{JE}^{-\alpha }P_{J}{\left|h_{JE}\right|}^{2}+\sigma_{E}^{2}\right). \end{array}$$

Therefore, the SINR for decoding ${\bar{x}}_{S}$ at $V_{E}$ is expressed as


$$\begin{array}{c} {\bar{\gamma }}_{E}^{{\bar{x}}_{S}}=\frac{\left(1-\ell \right)w_{S}\chi_{UE}P_{b}{\left|h_{UE}\right|}^{2}}{\left(1-\ell \right)\left(\varsigma_{0}P_{b}{\left|h_{UE}\right|}^{2}+d_{JE}^{-\alpha }P_{J}{\left|h_{JE}\right|}^{2}+\sigma_{E}^{2}\right)}. \end{array}$$ (19)

**Remark 2.** *In our analysis, we assume eavesdropper vehicle* $V_{E}$ *successfully decodes the common message and intercepts the covert message in a worst-case scenario. This methodology establishes a strict lower bound on the system’s security. Furthermore, modeling the jammer’s power as uniformly distributed provides a mathematically tractable approach to represent power uncertainty without requiring full channel state information at the jammer. The node positions are assumed fixed within a given communication block, adhering to the quasi-static fading assumption common in vehicular network modeling.*

## 3 Performance analysis

In this section, we analyze the reliability, security, and covertness performance of the proposed UAV-enabled RSMA vehicular network. Specifically, closed-form expressions for the OPs, IPs, asymptotic OPs, and sum throughput, as well as an analytical approximation for the DEP, are derived to characterize the overall system performance.

### 3.1 Outage probability analysis

The OP is adopted to evaluate the reliability performance of the considered system. An outage event occurs when a user fails to successfully decode its intended message, which is equivalent to the case where the received SINR falls below a predefined decoding threshold [34]. Mathematically, the OP is defined as


$$\begin{array}{c} O=\mathrm{Pr}\left(\bar{\gamma }<\varepsilon_{th}\right), \end{array}$$ (20)

where $\bar{\gamma }$ denotes the received SINR, $\varepsilon_{th}=2^{R_{th}}-1$ represents the SINR threshold, and $R_{th}$ denotes the target transmission rate.

#### 3.1.1 Outage probability at $V_{S}$.

When the transmitted signal does not contain the covert message ${\bar{x}}_{S}$ (i.e., $s_{b}=s_{b0}$ under hypothesis $H_{0}$), vehicle $V_{S}$ only needs to decode the common message. In this case, an outage event occurs when the common message ${\bar{x}}_{c}$ cannot be successfully decoded. Accordingly, the outage probability at $V_{S}$ is expressed as


$$\begin{array}{cc} O_{V_{S}} & =\mathrm{Pr}\left({\bar{\gamma }}_{S}^{{\bar{x}}_{c}}<\varepsilon_{th,S}^{c}\right)\\ & =1-\mathrm{Pr}\left({\bar{\gamma }}_{S}^{{\bar{x}}_{c}}>\varepsilon_{th,S}^{c}\right), \end{array}$$ (21)

where $\varepsilon_{th,S}^{c}=2^{R_{th,S}^{c}}-1$ denotes the SINR threshold required for decoding the common message, and $R_{th,S}^{c}$ represents the corresponding target transmission rate.

**Proposition 1.** *When* $s_{b}$ *does not contain* ${\bar{x}}_{S}$ *(i.e.,* $s_{b}=s_{b0}$*), the OP* $O_{V_{S}}$ *is given by (22) at the top of the next page, where*


$$\begin{array}{c} \Phi_{n,l}^{\left(1\right)}=\{\begin{array}{cc} \frac{{\left(G_{c,US}\eta d_{JS}^{-\alpha }{\bar{\rho }}_{J}+\mu_{JS}\right)}^{l-n}-\mu_{JS}^{l-n}}{l-n}, & l\neq n,\\ \ln\left(1+\frac{G_{c,US}\eta d_{JS}^{-\alpha }{\bar{\rho }}_{J}}{\mu_{JS}}\right), & l=n, \end{array} \end{array}$$

$\phi_{1}=w_{c}-\varepsilon_{th,S}^{c}\left(w_{S}+w_{I}\right)$, $\varphi_{c,US}=\frac{\varepsilon_{th,S}^{c}}{\chi_{US}\phi_{1}}$ *and* $G_{c,US}=\frac{\mu_{US}\varphi_{c,US}}{\rho_{b}}$.


$$\begin{array}{c} O_{V_{S}|H_{0}}=\{\begin{array}{ccc} & \begin{array}{cc} & 1-\frac{e^{-G_{c,US}}}{{\bar{\rho }}_{J}\Gamma \left(m_{JS}\right)}\sum_{t=0}^{m_{US}-1}\sum_{n=0}^{t}\sum_{l=0}^{n}\left(\begin{array}{c} t\\ n \end{array}\right)\left(\begin{array}{c} n\\ l \end{array}\right)\frac{{\left(-1\right)}^{n-l}G_{c,US}^{t-n-1}\mu_{JS}^{m_{JS}+n-l}}{t!\eta d_{JS}^{-\alpha }\Gamma {\left(m_{JS}+n\right)}^{-1}}\\ & \times \left[\frac{{\left(G_{c,US}\eta d_{JS}^{-\alpha }{\bar{\rho }}_{J}+\mu_{JS}\right)}^{l-m_{JS}-n+1}-\mu_{JS}^{l-m_{JS}-n+1}}{l-m_{JS}-n+1}\right], \end{array} & m_{JS}\neq 1,\\ & 1-\frac{e^{-G_{c,US}}}{{\bar{\rho }}_{J}}\sum_{t=0}^{m_{US}-1}\sum_{n=0}^{t}\sum_{l=0}^{n}\left(\begin{array}{c} t\\ n \end{array}\right)\left(\begin{array}{c} n\\ l \end{array}\right)\frac{{\left(-1\right)}^{n-l}G_{c,US}^{t-n-1}\mu_{JS}^{n+1-l}\Gamma \left(n+1\right)}{t!\eta d_{JS}^{-\alpha }}\Phi_{n,l}^{\left(1\right)}, & m_{JS}=1, \end{array} \end{array}$$ (22)

**Proof 1** *Please refer to Appendix A in* S1 Appendix*.*

To provide further insights into the reliability of the considered system, we investigate the asymptotic OP in the high-SNR regime. The diversity order characterizes how fast the outage probability decays as the transmit SNR increases and is defined as


$$\begin{array}{c} D_{V_{S}}=-\underset{\rho_{b}\to \infty }{\lim}\frac{\log\left(O_{V_{S}}\left(\rho_{b}\right)\right)}{\log\rho_{b}}. \end{array}$$ (23)

**Proposition 2.** *The asymptotic OP of* $V_{S}$ *in the high-SNR region with a uniformly distributed jammer power is given by*


$$\begin{array}{c} O_{V_{S}|H_{0}}^{\infty }=\frac{G_{c,US}^{m_{US}}}{m_{US}!}\sum_{k=0}^{m_{US}}\left(\begin{array}{c} m_{US}\\ k \end{array}\right)\frac{\eta^{k}d_{JS}^{-k\alpha }{\bar{\rho }}_{J}^{k}\Gamma (m_{JS}+k)}{\left(k+1\right)\Gamma (m_{JS})\mu_{JS}^{k}}. \end{array}$$ (24)

**Proof 2** *Please refer to Appendix B in* S1 Appendix*.*

Plugging (24) into (23), the diversity order of $V_{S}$ for the proposed system can be readily obtained as $D_{V_{S}|H_{0}}=m_{US}$.

When $s_{b}$ contains the covert message ${\bar{x}}_{S}$ (i.e., under hypothesis $H_{1}$ where $s_{b}=s_{b1}$), the OP of $V_{S}$ is characterized by two distinct failure events: i) $V_{S}$ fails to decode ${\bar{x}}_{c}$, and ii) $V_{S}$ successfully decodes ${\bar{x}}_{c}$ but fails to decode ${\bar{x}}_{S}$. Equivalently, this OP can be expressed as the complementary probability that $V_{S}$ successfully decodes both ${\bar{x}}_{c}$ and ${\bar{x}}_{S}$ simultaneously. Therefore, the OP is formulated as


$$\begin{array}{c} O_{V_{S}}=1-\mathrm{Pr}\left({\bar{\gamma }}_{S}^{{\bar{x}}_{c}}>\varepsilon_{th,S}^{c},{\bar{\gamma }}_{S}^{{\bar{x}}_{S}}>\varepsilon_{th,S}^{S}\right), \end{array}$$ (25)

where $\varepsilon_{th,S}^{S}=2^{R_{th,S}^{S}}-1$ denotes the SINR threshold required for decoding ${\bar{x}}_{S}$, and $R_{th,S}^{S}$ represents the corresponding target data rate.

Under hypothesis $H_{1}$ (i.e., $s_{b}=s_{b1}$), and following a derivation procedure similar to that used for (22), the closed-form expression of $O_{V_{S}}$ is obtained as (26), shown at the top of the next page, where


$$\begin{array}{c} \Phi_{n,l}^{\left(2\right)}=\{\begin{array}{cc} \frac{{\left(G_{\max,US}\eta d_{JS}^{-\alpha }{\bar{\rho }}_{J}+\mu_{JS}\right)}^{l-n}-\mu_{JS}^{l-n}}{l-n}, & l\neq n,\\ \ln\left(1+\frac{G_{\max,US}\eta d_{JS}^{-\alpha }{\bar{\rho }}_{J}}{\mu_{JS}}\right), & l=n, \end{array} \end{array}$$

$\phi_{2}=w_{c}-\varepsilon_{th,S}^{S}w_{S}$, $\varphi_{S,US}=\frac{\varepsilon_{th,S}^{S}}{\chi_{US}\phi_{2}}$, $G_{S,US}=\frac{\mu_{US}\varphi_{S,US}}{\rho_{b}}$ and $G_{\max,US}=\max\left(G_{c,US},G_{S,US}\right)$.


$$\begin{array}{c} O_{V_{S}|H_{1}}=\{\begin{array}{ccc} & \begin{array}{cc} & 1-\frac{e^{-G_{\max,US}}}{{\bar{\rho }}_{J}\Gamma \left(m_{JS}\right)}\sum_{t=0}^{m_{US}-1}\sum_{n=0}^{t}\sum_{l=0}^{n}\left(\begin{array}{c} t\\ n \end{array}\right)\left(\begin{array}{c} n\\ l \end{array}\right)\frac{{\left(-1\right)}^{n-l}G_{\max,US}^{t-n-1}\mu_{JS}^{m_{JS}+n-l}}{t!\eta d_{JS}^{-\alpha }\Gamma {\left(m_{JS}+n\right)}^{-1}}\\ & \times \left[\frac{{\left(G_{\max,US}\eta d_{JS}^{-\alpha }{\bar{\rho }}_{J}+\mu_{JS}\right)}^{l-m_{JS}-n+1}-\mu_{JS}^{l-m_{JS}-n+1}}{l-m_{JS}-n+1}\right], \end{array} & m_{JS}\neq 1,\\ & 1-\frac{e^{-G_{\max,US}}}{{\bar{\rho }}_{J}}\sum_{t=0}^{m_{US}-1}\sum_{n=0}^{t}\sum_{l=0}^{n}\left(\begin{array}{c} t\\ n \end{array}\right)\left(\begin{array}{c} n\\ l \end{array}\right)\frac{{\left(-1\right)}^{n-l}G_{\max,US}^{t-n-1}\mu_{JS}^{n+1-l}\Gamma \left(n+1\right)}{t!\eta d_{JS}^{-\alpha }}\Phi_{n,l}^{\left(2\right)}, & m_{JS}=1, \end{array} \end{array}$$ (26)

Based on (26), the asymptotic outage behavior of $O_{V_{S}|H_{1}}$ can be derived in the high-SNR regime, i.e., when $\rho_{b}\to \infty$. The corresponding asymptotic expression is given by


$$\begin{array}{c} O_{V_{S}|H_{1}}^{\infty }=\frac{G_{\max,US}^{m_{US}}}{m_{US}!}\sum_{k=0}^{m_{US}}\left(\begin{array}{c} m_{US}\\ k \end{array}\right)\frac{\eta^{k}d_{JS}^{-k\alpha }{\bar{\rho }}_{J}^{k}\Gamma \left(m_{JS}+k\right)}{\left(k+1\right)\Gamma \left(m_{JS}\right)\mu_{JS}^{k}}. \end{array}$$ (27)

By substituting (27) into (23), the diversity order of $V_{S}$ under hypothesis $H_{1}$ is obtained as $D_{V_{S}|H_{1}}=m_{US}$.

#### 3.1.2 Outage probability at $V_{I}$.

Regardless of whether $s_{b}$ contains ${\bar{x}}_{S}$, the signal ${\bar{x}}_{I}$ is always transmitted. Hence, $V_{I}$ must successfully decode both the common and its intended private information to avoid an outage event. Accordingly, the OP at $V_{I}$ is defined as


$$\begin{array}{c} O_{V_{I}}=1-\mathrm{Pr}\left({\bar{\gamma }}_{I}^{{\bar{x}}_{c}}>\varepsilon_{th,I}^{c},{\bar{\gamma }}_{I}^{{\bar{x}}_{I}}>\varepsilon_{th,I}^{I}\right), \end{array}$$ (28)

where $\varepsilon_{th,I}^{c}=2^{R_{th,I}^{c}}-1$ and $\varepsilon_{th,I}^{I}=2^{R_{th,I}^{I}}-1$ denote the SINR thresholds required for decoding ${\bar{x}}_{c}$ and ${\bar{x}}_{I}$, respectively, while $R_{th,I}^{c}$ and $R_{th,I}^{I}$ represent the corresponding target data rates.

By following a derivation procedure similar to that used for obtaining $O_{V_{S}|H_{1}}$, the closed-form expression of $O_{V_{I}}$ can be derived as (29), shown at the top of the next page, where

$\Phi_{n,l}^{\left(3\right)}=\{\begin{array}{cc} \frac{{\left(G_{\max,UI}\eta d_{JI}^{-\alpha }{\bar{\rho }}_{J}+\mu_{JI}\right)}^{l-n}-\mu_{JI}^{l-n}}{l-n}, & l\neq n,\\ \ln\left(1+\frac{G_{\max,UI}\eta d_{JI}^{-\alpha }{\bar{\rho }}_{J}}{\mu_{JI}}\right), & l=n, \end{array}$,

$\phi_{3}=w_{c}-\varepsilon_{th,I}^{c}\left(w_{S}+w_{I}\right)$, $\phi_{4}=w_{I}-\varepsilon_{th,I}^{I}w_{S}$, $\varphi_{c,UI}=\frac{\varepsilon_{th,I}^{c}}{\chi_{UI}\phi_{3}}$, $\varphi_{I,UI}=\frac{\varepsilon_{th,I}^{I}}{\chi_{UI}\phi_{4}}$, $G_{c,UI}=\frac{\mu_{UI}\varphi_{c,UI}}{\rho_{b}}$, $G_{I,UI}=\frac{\mu_{UI}\varphi_{I,UI}}{\rho_{b}}$ and $G_{\max,UI}=\max\left(G_{c,UI},G_{I,UI}\right)$.


$$\begin{array}{c} O_{V_{I}}=\{\begin{array}{ccc} & \begin{array}{cc} & 1-\frac{e^{-G_{\max,UI}}}{{\bar{\rho }}_{J}\Gamma \left(m_{JI}\right)}\sum_{t=0}^{m_{UI}-1}\sum_{n=0}^{t}\sum_{l=0}^{n}\left(\begin{array}{c} t\\ n \end{array}\right)\left(\begin{array}{c} n\\ l \end{array}\right)\frac{{\left(-1\right)}^{n-l}G_{\max,UI}^{t-n-1}\mu_{JI}^{m_{JI}+n-l}}{t!\eta d_{JI}^{-\alpha }\Gamma {\left(m_{JI}+n\right)}^{-1}}\\ & \times \left[\frac{{\left(G_{\max,UI}\eta d_{JI}^{-\alpha }{\bar{\rho }}_{J}+\mu_{JI}\right)}^{l-m_{JI}-n+1}-\mu_{JI}^{l-m_{JI}-n+1}}{l-m_{JI}-n+1}\right], \end{array} & m_{JI}\neq 1,\\ & 1-\frac{e^{-G_{\max,UI}}}{{\bar{\rho }}_{J}}\sum_{t=0}^{m_{UI}-1}\sum_{n=0}^{t}\sum_{l=0}^{n}\left(\begin{array}{c} t\\ n \end{array}\right)\left(\begin{array}{c} n\\ l \end{array}\right)\frac{{\left(-1\right)}^{n-l}G_{\max,UI}^{t-n-1}\mu_{JI}^{n+1-l}\Gamma \left(n+1\right)}{t!\eta d_{JI}^{-\alpha }}\Phi_{n,l}^{\left(3\right)}, & m_{JI}=1, \end{array} \end{array}$$ (29)

We next derive the asymptotic expression of $O_{V_{I}}$ when the transmit SNR tends to infinity, i.e., $\rho_{b}\to \infty$. The result is expressed as


$$\begin{array}{c} O_{V_{I}}^{\infty }=\frac{G_{\max,UI}^{m_{UI}}}{m_{UI}!}\sum_{k=0}^{m_{UI}}\left(\begin{array}{c} m_{UI}\\ k \end{array}\right)\frac{\eta^{k}d_{JI}^{-k\alpha }{\bar{\rho }}_{J}^{k}\Gamma \left(m_{JI}+k\right)}{\left(k+1\right)\Gamma \left(m_{JI}\right)\mu_{JI}^{k}}. \end{array}$$ (30)

By substituting (30) into (23), the diversity order of $V_{I}$ for the proposed system is obtained as $D_{V_{I}}=m_{UI}$.

**Remark 3.** *It can be observed that the OPs of both* $V_{S}$ *and* $V_{I}$ *decrease as the transmit SNR* $\rho_{b}$ *increases. In the high-SNR regime, the slopes of the OP curves are dominated by the fading parameters, indicating that the achievable diversity orders of the proposed system are governed by the Nakagami-m parameters of the corresponding communication links.*

The sum throughput of the system can be calculated as [42, Eq. (13)]


$$\begin{array}{c} {𝕋}_{sum}=\frac{\left(1-O_{V_{S}}\right)\left(R_{th,S}^{c}+R_{th,S}^{S}\right)}{2}+\frac{\left(1-O_{V_{I}}\right)\left(R_{th,I}^{c}+R_{th,I}^{I}\right)}{2}. \end{array}$$ (31)

### 3.2 Performance analysis of $V_{E}$

According to the operational mechanism of $V_{E}$, the eavesdropper vehicle first attempts to decode the common signal ${\bar{x}}_{c}$ to facili\tate subsequent monitoring. After successfully decoding and removing ${\bar{x}}_{c}$ via SIC, $V_{E}$ allocates ℓ and 1−ℓ portions of the remaining power ${\hat{P}}_{E}$ to monitor and decode ${\bar{x}}_{S}$, respectively. Accordingly, three performance metrics are evaluated: the IP for eavesdropping on the common signal ${\bar{x}}_{c}$, the DEP for monitoring the presence of ${\bar{x}}_{S}$, and the IP for eavesdropping on ${\bar{x}}_{S}$ after monitoring.

#### 3.2.1 Intercept probability of $V_{E}$ on ${\bar{x}}_{c}$.

The intercept probability is defined as the probability that $V_{E}$ successfully decodes the target signal. Hence, the IP of $V_{E}$ when intercepting ${\bar{x}}_{c}$ is given by


$$\begin{array}{c} I_{V_{E}|{\bar{x}}_{c}}=\mathrm{Pr}\left({\bar{\gamma }}_{E}^{{\bar{x}}_{c}}>\varepsilon_{th,E}^{c}\right), \end{array}$$ (32)

where $\varepsilon_{th,E}^{c}=2^{R_{th,E}^{c}}-1$ denotes the SINR threshold required to decode ${\bar{x}}_{c}$, and $R_{th,E}^{c}$ represents the corresponding target rate.

Based on (14) and (17), it can be observed that the SINR of $V_{E}$ when intercepting ${\bar{x}}_{c}$ depends on whether the private signal ${\bar{x}}_{S}$ is transmitted (i.e., $w_{S}\neq 0$). For analytical convenience, a unified expression is provided in the following (33),


$$\begin{array}{c} I_{V_{E}|{\bar{x}}_{c}}=\{\begin{array}{ccc} & \begin{array}{cc} & \frac{e^{-G_{c,UE}}}{{\bar{\rho }}_{J}\Gamma \left(m_{JE}\right)}\sum_{t=0}^{m_{UE}-1}\sum_{n=0}^{t}\sum_{l=0}^{n}\left(\begin{array}{c} t\\ n \end{array}\right)\left(\begin{array}{c} n\\ l \end{array}\right)\frac{{\left(-1\right)}^{n-l}G_{c,UE}^{t-n-1}\mu_{JE}^{m_{JE}+n-l}}{t!d_{JE}^{-\alpha }\Gamma {\left(m_{JE}+n\right)}^{-1}}\\ & \times \left[\frac{{\left(G_{c,UE}d_{JE}^{-\alpha }{\bar{\rho }}_{J}+\mu_{JE}\right)}^{l-m_{JE}-n+1}-\mu_{JE}^{l-m_{JE}-n+1}}{l-m_{JE}-n+1}\right], \end{array} & m_{JE}\neq 1,\\ & \frac{e^{-G_{c,UE}}}{{\bar{\rho }}_{J}}\sum_{t=0}^{m_{UE}-1}\sum_{n=0}^{t}\sum_{l=0}^{n}\left(\begin{array}{c} t\\ n \end{array}\right)\left(\begin{array}{c} n\\ l \end{array}\right)\frac{{\left(-1\right)}^{n-l}G_{c,UE}^{t-n-1}\mu_{JE}^{n+1-l}\Gamma \left(n+1\right)}{t!d_{JE}^{-\alpha }}\Phi_{n,l}^{\left(4\right)}, & m_{JE}=1, \end{array} \end{array}$$ (33)

in which


$$\begin{array}{c} \Phi_{n,l}^{\left(4\right)}=\{\begin{array}{cc} \frac{{\left(G_{c,UE}d_{JE}^{-\alpha }{\bar{\rho }}_{J}+\mu_{JE}\right)}^{l-n}-\mu_{JE}^{l-n}}{l-n}, & l\neq n,\\ \ln\left(1+\frac{G_{c,UE}d_{JE}^{-\alpha }{\bar{\rho }}_{J}}{\mu_{JE}}\right), & l=n, \end{array} \end{array}$$

$\phi_{5}=w_{c}-\varepsilon_{th,E}^{c}\left(w_{S}+w_{I}\right)$, $\varphi_{c,UE}=\frac{\varepsilon_{th,E}^{c}}{\chi_{UE}\phi_{5}}$ and $G_{c,UE}=\frac{\mu_{UE}\varphi_{c,UE}}{\rho_{b}}$.

#### 3.2.2 Detection error probability for monitoring ${\bar{x}}_{S}$.

To determine whether the covert message ${\bar{x}}_{S}$ exists, $V_{E}$ performs a binary hypothesis test based on the received signal. Specifically, the monitoring process is formulated as a covert detection problem, in which $V_{E}$ decides between two hypotheses corresponding to the absence or presence of ${\bar{x}}_{S}$. The detection performance is evaluated through the DEP, which characterizes the reliability of the monitoring process.


$$\begin{array}{c} DEP\left(\tau \right)=p_{f}+p_{m}, \end{array}$$ (34)

where $p_{f}=\mathrm{Pr}\left(D_{1}|H_{0}\right)$ and $p_{m}=\mathrm{Pr}\left(D_{0}|H_{1}\right)$ denote the *false alarm probability* and *miss detection probability*, respectively. More specifically, $p_{f}$ represents the probability that $V_{E}$ incorrectly declares the existence of ${\bar{x}}_{S}$ under the hypothesis $H_{0}$ (i.e., ${\bar{x}}_{S}$ is absent), whereas $p_{m}$ denotes the probability that $V_{E}$ fails to detect ${\bar{x}}_{S}$ under the hypothesis $H_{1}$ (i.e., ${\bar{x}}_{S}$ is present). Therefore, DEP(τ) reflects the overall monitoring reliability of $V_{E}$ with respect to the detection threshold τ. In particular, DEP(τ)=0 indicates perfect monitoring performance, while DEP(τ)=1 means that $V_{E}$ cannot detect the covert signal.

**Proposition 3.** *The false alarm probability (*$p_{f}$*) at* $V_{E}$ *can be expressed as*


pf≈π2K∑k=1K|sin((2k−1)π2K)|[μJEmJEe−ΛμUEς0ρbΓ(mJE)∑t=0mUE−1∑l=0t×(tl)(−1)lΛt−lμUEtψklt!ς0tρbtdJElαG(ψk)mJE+lγ(mJE+l,G(ψk)ΛdJE−αψk)+Γ(mJE,μJEΛ/μJEΛdJE−αψkdJE−αψk)Γ(mJE)], (35)

*where* $G\left(x\right)=\mu_{JE}-\frac{\mu_{UE}d_{JE}^{-\alpha }x}{\varsigma_{0}\rho_{b}}$, $\xi_{k}=\cos\left(\frac{2k-1}{2K}\pi \right)$, $\psi_{k}=\frac{{\bar{\rho }}_{J}\left(\xi_{k}+1\right)}{2}$ *and K is a complexity-accuracy tradeoff parameter.*

**Proof 3** *Please refer to Appendix C in* S1 Appendix*.*

By adopting a similar approach, the miss detection probability $p_{m}$ is given by


pm≈1−π2K∑k=1K|sin((2k−1)π2K)|[μJEmJEe−ΛμUEς1ρbΓ(mJE)∑t=0mUE−1∑l=0t×(tl)(−1)lΛt−lμUEtψklt!ς1tρbtdJElαΔ(ψk)mJE+lγ(mJE+l,Δ(ψk)ΛdJE−αψk)+Γ(mJE,μJEΛ/μJEΛdJE−αψkdJE−αψk)Γ(mJE)], (36)

where $\Delta \left(x\right)=\mu_{JE}-\frac{\mu_{UE}d_{JE}^{-\alpha }x}{\varsigma_{1}\rho_{b}}$.

By substituting (35) and (36) into (34), an analytical approximation of the DEP is obtained.

The IP of $V_{E}$ when attempting to intercept ${\bar{x}}_{S}$ is defined as


$$\begin{array}{c} I_{V_{E}|{\bar{x}}_{S}}=\mathrm{Pr}\left({\bar{\gamma }}_{E}^{{\bar{x}}_{S}}>\varepsilon_{th,E}^{S}\right), \end{array}$$ (37)

where $\varepsilon_{th,E}^{S}=2^{R_{th,E}^{S}}-1$ denotes the SINR threshold for decoding ${\bar{x}}_{S}$, and $R_{th,E}^{S}$ is the corresponding target rate.

By substituting the SINR expression given in (19) into (37), the IP is obtained as


$$\begin{array}{c} I_{V_{E}|{\bar{x}}_{S}}=\{\begin{array}{ccc} & \begin{array}{cc} & \frac{e^{-G_{S,UE}}}{{\bar{\rho }}_{J}\Gamma \left(m_{JE}\right)}\sum_{t=0}^{m_{UE}-1}\sum_{n=0}^{t}\sum_{l=0}^{n}\left(\begin{array}{c} t\\ n \end{array}\right)\left(\begin{array}{c} n\\ l \end{array}\right)\frac{{\left(-1\right)}^{n-l}G_{S,UE}^{t-n-1}\mu_{JE}^{m_{JE}+n-l}}{t!d_{JE}^{-\alpha }\Gamma {\left(m_{JE}+n\right)}^{-1}}\\ & \times \left[\frac{{\left(G_{S,UE}d_{JE}^{-\alpha }{\bar{\rho }}_{J}+\mu_{JE}\right)}^{l-m_{JE}-n+1}-\mu_{JE}^{l-m_{JE}-n+1}}{l-m_{JE}-n+1}\right], \end{array} & m_{JE}\neq 1,\\ & \frac{e^{-G_{S,UE}}}{{\bar{\rho }}_{J}}\sum_{t=0}^{m_{UE}-1}\sum_{n=0}^{t}\sum_{l=0}^{n}\left(\begin{array}{c} t\\ n \end{array}\right)\left(\begin{array}{c} n\\ l \end{array}\right)\frac{{\left(-1\right)}^{n-l}G_{S,UE}^{t-n-1}\mu_{JE}^{n+1-l}\Gamma \left(n+1\right)}{t!d_{JE}^{-\alpha }}\Phi_{n,l}^{\left(5\right)}, & m_{JE}=1, \end{array} \end{array}$$ (38)

where $\varphi_{S,UE}=\frac{\varepsilon_{th,E}^{S}}{w_{S}\chi_{UE}-\varepsilon_{th,E}^{S}\varsigma_{0}}$, $G_{S,UE}=\frac{\mu_{UE}\varphi_{S,UE}}{\rho_{b}}$ and


$$\begin{array}{c} \Phi_{n,l}^{\left(5\right)}=\{\begin{array}{cc} \frac{{\left(G_{S,UE}d_{JE}^{-\alpha }{\bar{\rho }}_{J}+\mu_{JE}\right)}^{l-n}-\mu_{JE}^{l-n}}{l-n}, & l\neq n,\\ \ln\left(1+\frac{G_{S,UE}d_{JE}^{-\alpha }{\bar{\rho }}_{J}}{\mu_{JE}}\right), & l=n, \end{array} \end{array}$$

**Remark 4.** *Although the derived closed-form expressions for IP and analytical approximation for DEP involve complex finite sums and special functions, they provide profound physical insights. From a practical system design perspective, these formulas explicitly reveal that the covertness performance is fundamentally governed by the jammer-to-eavesdropper distance (*$d_{JE}$*) and the random jamming power bounds (*${\bar{\rho }}_{J}$*). This allows network designers to quantitatively determine the minimum required jamming power to guarantee a target covertness level without conducting time-consuming field measurements.*

## 4 Numerical results

This section presents numerical and simulation results to validate the analytical expressions of OP, DEP, IP, and system throughput for the proposed UAV-assisted covert transportation communication system. Unless otherwise specified, all wireless links are assumed to experience Nakagami-*m* fading, where the fading parameters are set as $m=m_{US}=m_{UI}=m_{UE}=m_{JS}=m_{JI}=m_{JE}$. The average channel power gains are assumed to be identical, i.e., $\lambda_{US}=\lambda_{UI}=\lambda_{UE}=\lambda_{JS}=\lambda_{JI}=\lambda_{JE}=1$. The final results were obtained using a Monte Carlo simulation over 10^6^ random channel realizations. The positions of the UAV, $V_{S}$, $V_{I}$, and $V_{E}$ are fixed at (0,0,100) m, (50,50,0) m, (10,50,0) m, and (20,50,0) m, respectively. To enhance transmission covertness, a vehicle-mounted jammer is considered, where the transmit power is randomly distributed according to a uniform distribution over $\left[0,{\bar{\rho }}_{J}\right]$. In the following figures, “Ana.”, “Sim.”, and “Asym.” denote analytical results, Monte Carlo simulations, and high-SNR asymptotic analysis, respectively. Furthermore, *K* = 100 is selected following [43] for the Gauss-Chebyshev quadrature parameter to guarantee rapid numerical convergence and ensure high accuracy between theoretical analytical curves and Monte Carlo simulations. The remaining system parameters used in the simulations are summarized in Table 2. To maintain the total power allocation constraint $w_{c}+w_{S}+w_{I}=1$, the power allocation factors for the private messages are generally set to $w_{S}=w_{I}=\frac{1-w_{c}}{2}$ across all simulation scenarios. Accordingly, when $w_{c}$ varies (e.g., in Figs 4, 6, and 8), $w_{S}$ and $w_{I}$ are dynamically adjusted to ensure equal power sharing for the two legitimate private transmissions.

**Table 2:** Simulation parameters.

Parameter	Value
$w_{c}$	0.5
η	1
$R_{th,S}^{c}$	0.3 bpcu
$R_{th,S}^{S}$	0.1 bpcu
$R_{th,I}^{c}$	0.2 bpcu
$R_{th,I}^{I}$	0.1 bpcu
$R_{th,E}^{c}$	0.2 bpcu
$R_{th,E}^{S}$	0.2 bpcu
α	2
ℓ	0.5
$w_{S}$	0.3
$w_{I}$	0.3
*c*	$3\times 10^{8}\text{ m/s}$
*f*	2 GHz
$d_{JS}$	25 m
$d_{JI}$	5 m
$d_{JE}$	10 m
*m*	{1,2,3,4}
${\bar{\rho }}_{J}$	10 dB
*K*	100

Fig 2 illustrates the OP performance of the legitimate vehicular users under different transmit SNR values. As expected, the OP gradually decreases as the transmit SNR increases, indicating that higher transmission power improves the reliability of data delivery in the UAV-assisted transportation network. It can also be observed that the analytical curves closely match the Monte Carlo simulation results, which verifies the accuracy of the derived closed-form expressions. Moreover, the high-SNR asymptotic results follow the same slope as the exact analytical curves in the high-SNR region, confirming the correctness of the diversity order analysis. Another noticeable observation is that the fading severity significantly affects system reliability, where larger Nakagami-*m* values lead to improved outage performance due to reduced channel fluctuations. These results collectively demonstrate that the proposed RSMA-based transmission framework effectively enhances communication robustness for vehicular users under covert communication constraints.

**Fig 2:**
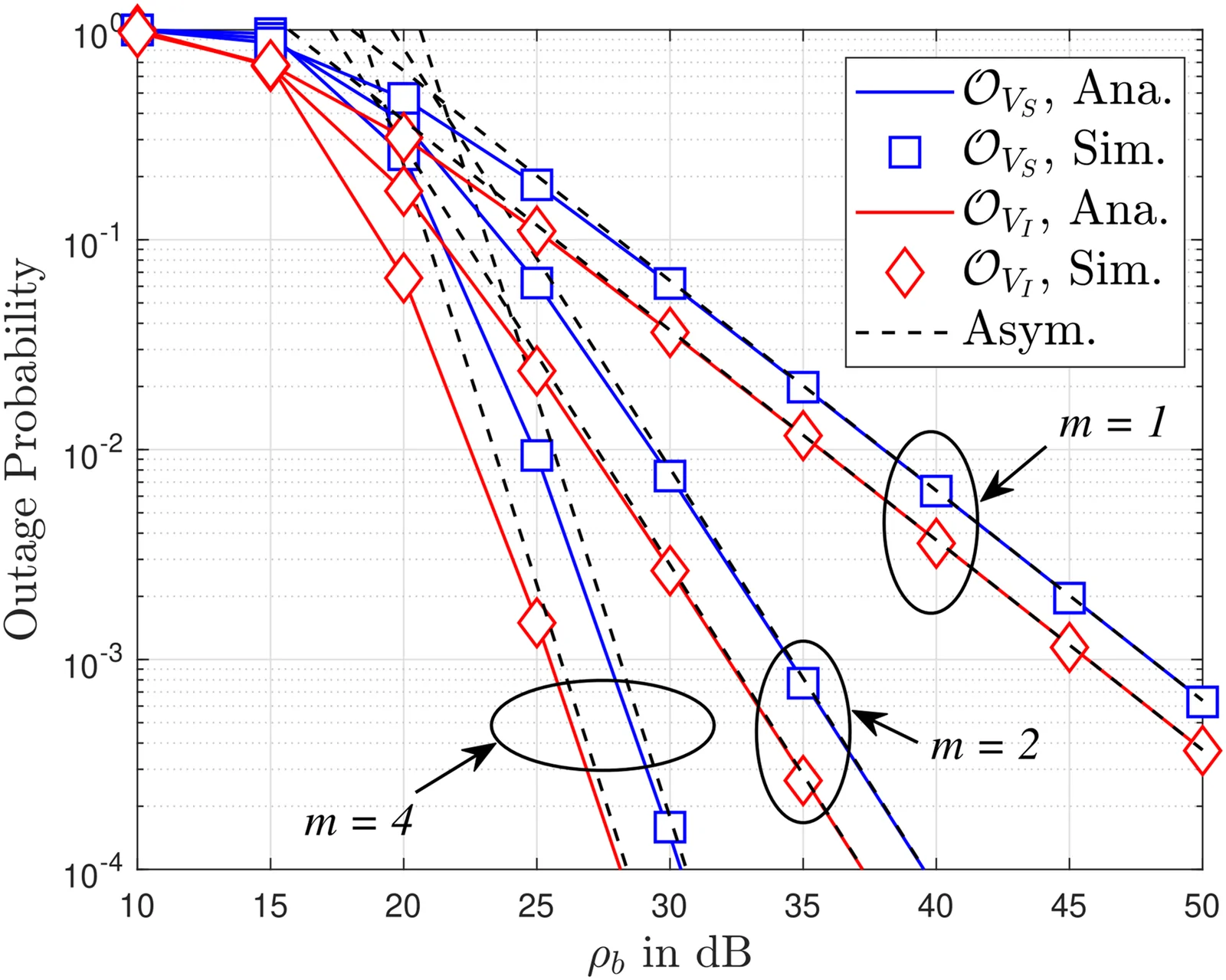
The OP versus $\rho_{b}$ in dB.

Fig 3 illustrates the impact of the average jamming power ${\bar{\rho }}_{J}$ on the outage performance of the vehicular users. It is observed that increasing ${\bar{\rho }}_{J}$ generally degrades the decoding capability of legitimate vehicles due to stronger interference introduced by the vehicle-mounted jammer. However, this degradation indirectly enhances communication covertness by increasing the uncertainty at the monitoring vehicle. This reveals a critical trade-off between communication reliability and covertness: while a higher maximum jamming power effectively masks the covert signal from the eavesdropper, it simultaneously deteriorates the legitimate transmission. Therefore, a careful design of the jamming power upper bound ${\bar{\rho }}_{J}$ is essential in practical UAV-assisted networks to maintain a target outage probability while maximizing covertness.

**Fig 3:**
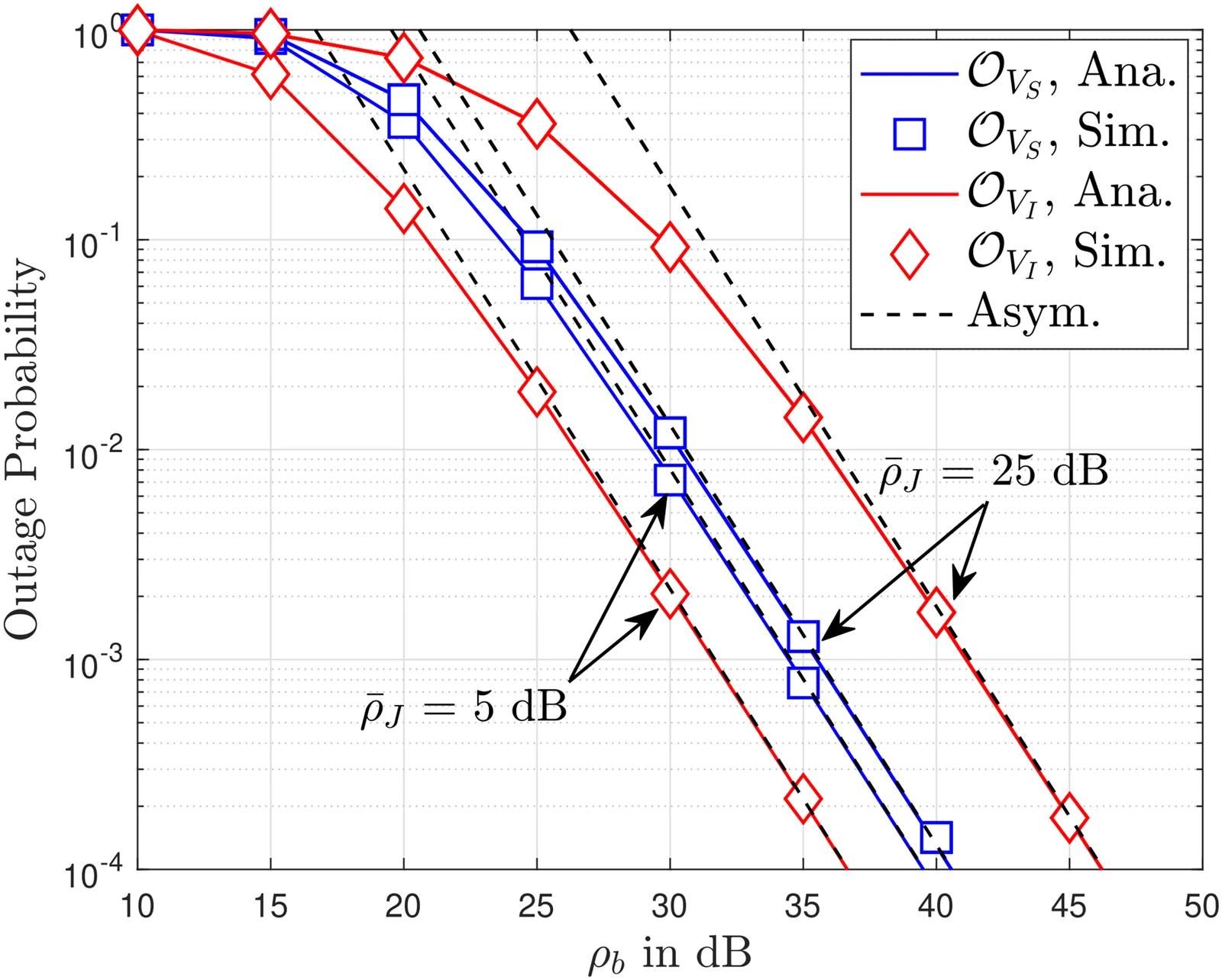
The OP versus $\rho_{b}$ in dB, with *m* = 2.

Fig 4 depicts the effect of the common message power allocation coefficient $w_{c}$ on the outage performance of vehicular users. It can be observed that increasing $w_{c}$ generally improves the decoding reliability of the common signal but reduces the power allocated to private messages, which may degrade the performance of individual users. This highlights the inherent power allocation trade-off in RSMA systems. From a practical design perspective, dynamically optimizing the power allocation coefficient $w_{c}$ based on the real-time channel conditions of $V_{S}$ and $V_{I}$ is crucial. An optimal $w_{c}$ must balance the successful SIC process with the decoding of private messages, ensuring fair and reliable service without leaking excessive information.

**Fig 4:**
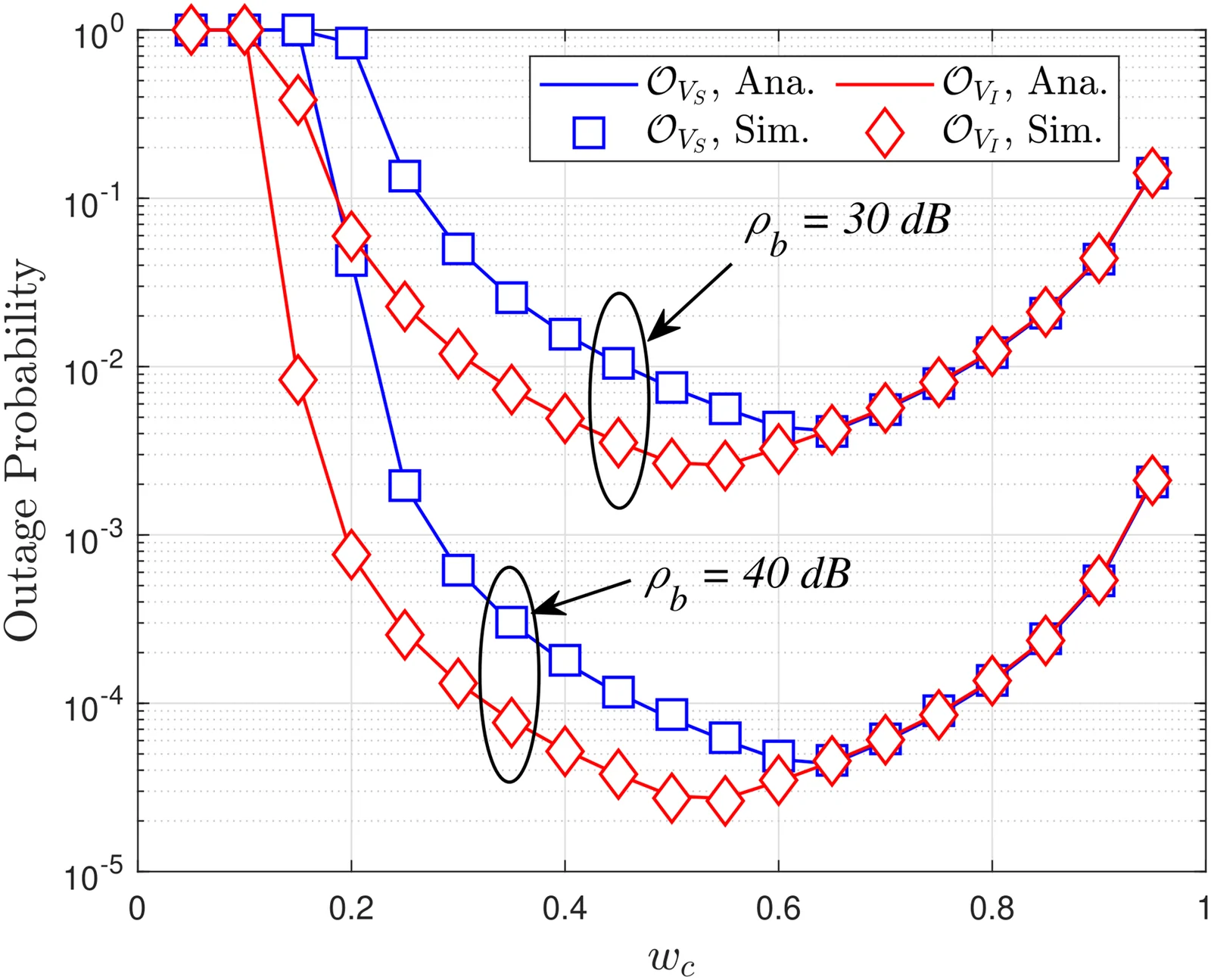
The OP versus $w_{c}$, with *m* = 2.

Fig 5 illustrates the sum throughput of the proposed system versus the horizontal location of the UAV, denoted by $x_{U}$. It can be observed that the system throughput varies noticeably with the UAV position due to changes in propagation distances between the UAV and ground vehicles. When the UAV moves closer to the legitimate vehicles, the received signal strength improves, leading to a higher achievable sum throughput. Conversely, when the UAV is located farther away, the increased path loss degrades transmission efficiency.

**Fig 5:**
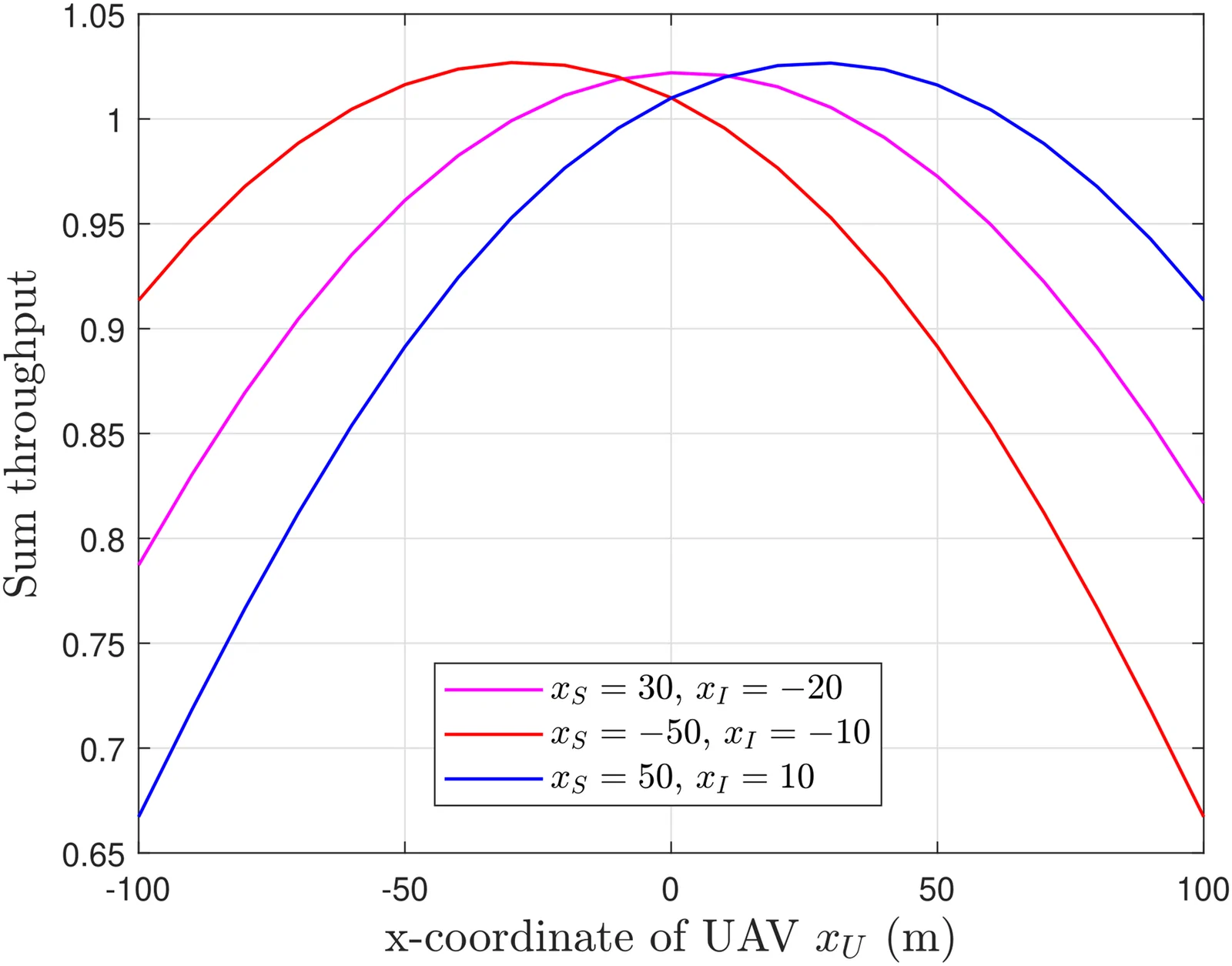
Sum throughput versus $x_{U}$, with *m* = 2, $R_{th,S}^{c}=R_{th,I}^{c}=0.7bps/Hz$, $R_{th,S}^{S}=R_{th,I}^{I}=0.5bps/Hz$ and $\rho_{b}=30dB$.

Fig 6 illustrates the IP of $V_{E}$ for the common message ${\bar{x}}_{c}$ versus the transmit SNR $\rho_{b}$ under different power allocation factors $w_{c}$. It can be observed that the IP increases with $\rho_{b}$ since higher transmit power improves the received signal strength at the monitoring vehicle, which makes the interception of ${\bar{x}}_{c}$ more likely. Furthermore, larger values of $w_{c}$ lead to higher IP because more transmit power is assigned to the common message, thereby enhancing its decodability at $V_{E}$. In contrast, smaller $w_{c}$ values reduce the exposure level of ${\bar{x}}_{c}$, thus improving covert performance.

**Fig 6:**
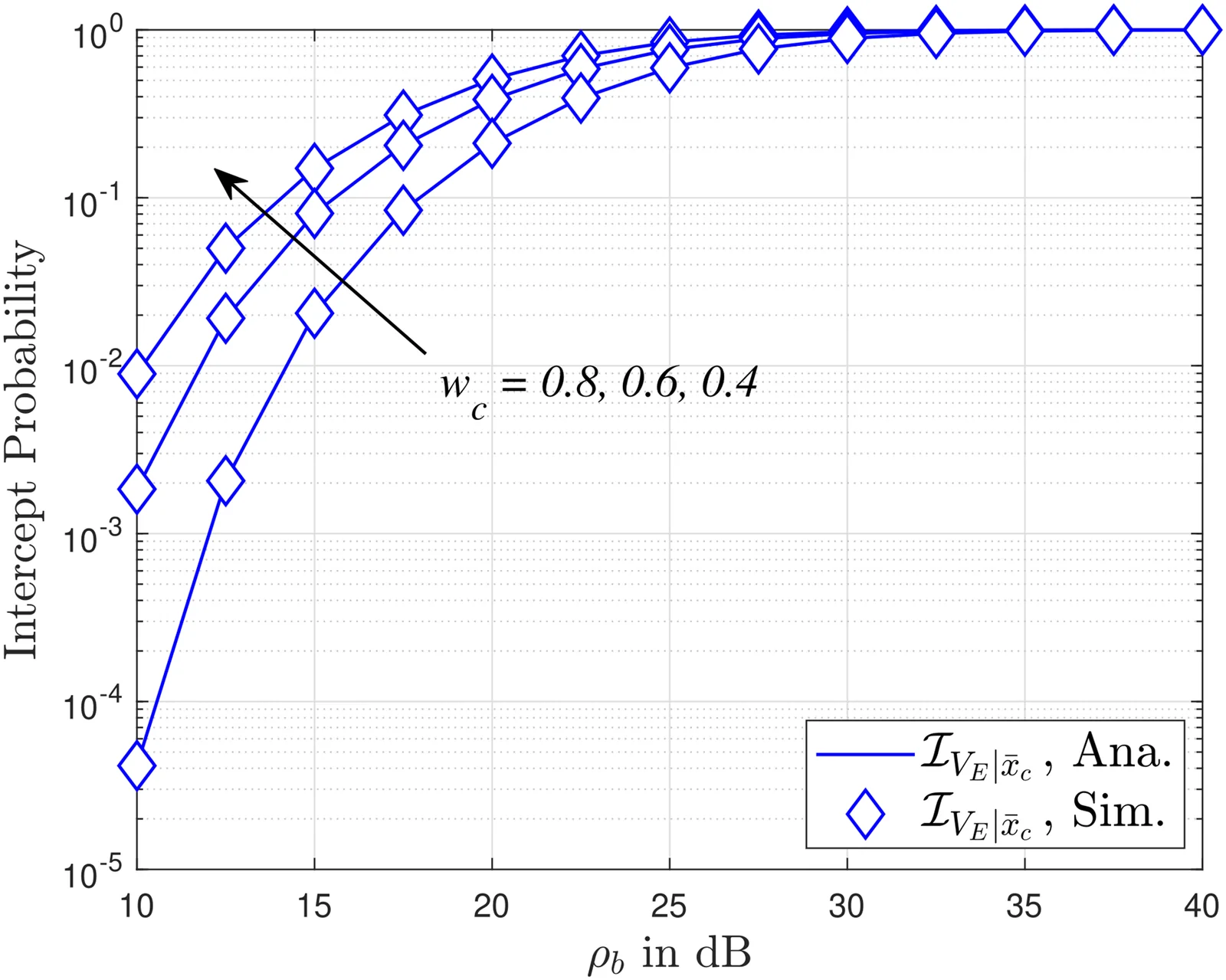
The IP versus $\rho_{b}$, with *m* = 2 and ${\bar{\rho }}_{J}=30dB$.

Fig 7 presents the DEP versus the detection threshold τ (in dB). It can be seen that the DEP initially decreases to a minimum value and then increases as τ varies. When τ is too small, the monitoring node tends to frequently declare the existence of covert transmission, resulting in a high false alarm rate. Conversely, when τ becomes excessively large, the probability of missed detection dominates, which again drives the DEP back up to 1. Therefore, there exists a specific threshold where the DEP reaches its minimum value, representing the worst-case covertness scenario for the legitimate system. The analytical curves match closely with the Monte Carlo simulations, which confirms the accuracy of the theoretical derivations.

**Fig 7:**
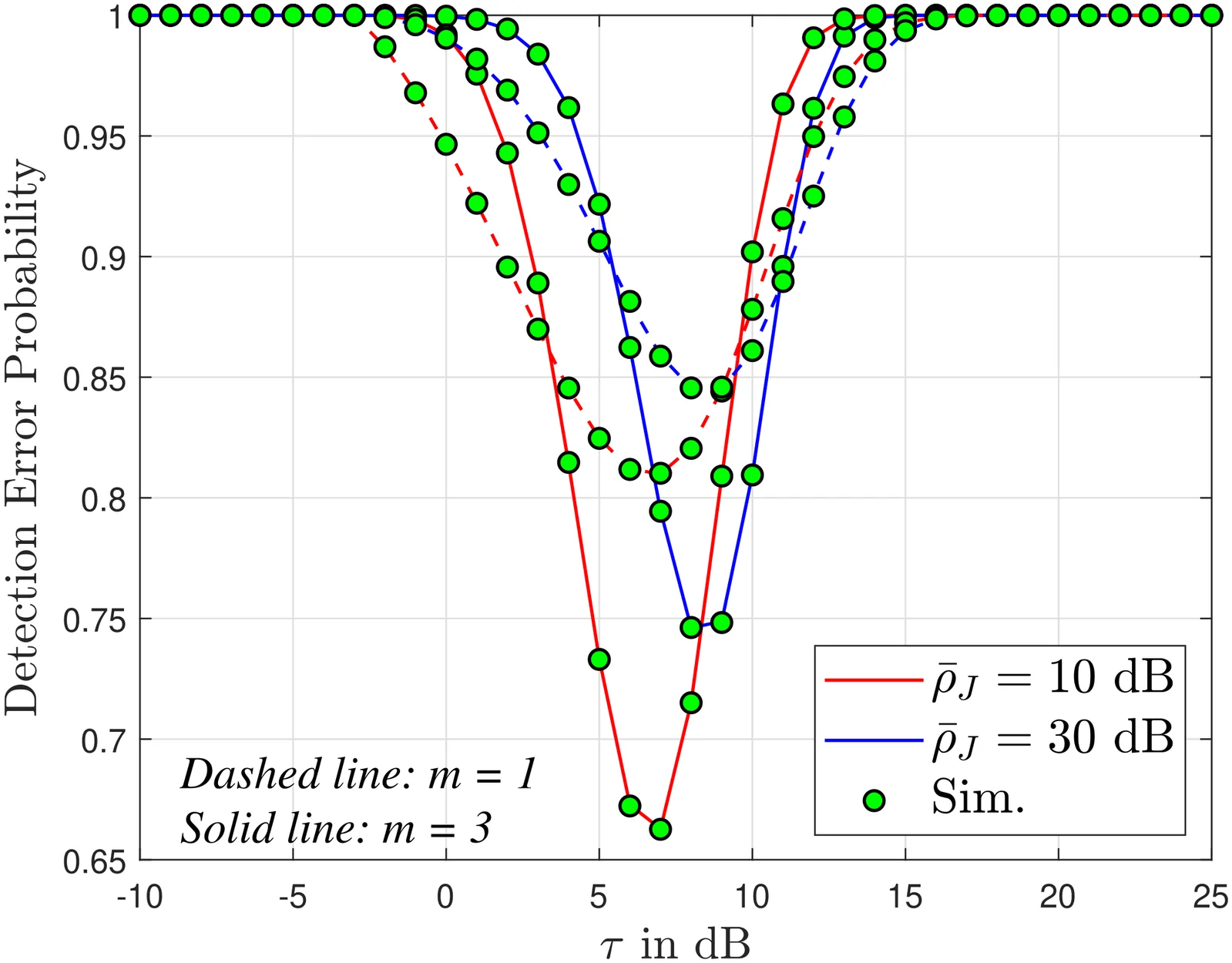
The DEP versus τ in dB, with $w_{c}=0.2$, ℓ=0.5 and $\rho_{b}=30dB$.

Fig 8 illustrates the DEP versus the power allocation factor $w_{c}$ for different detection thresholds τ and jamming power levels ${\bar{\rho }}_{J}$. It is observed that the DEP generally increases with larger $w_{c}$, implying that allocating more power to the common signal can improve covertness performance. This behavior occurs because increasing $w_{c}$ reduces the relative power assigned to the covert signal, thereby making the monitoring process at $V_{E}$ more uncertain. Additionally, varying the detection threshold τ shifts the DEP curves owing to the modified decision criteria at $V_{E}$. However, excessively large $w_{c}$ may degrade the reliability of private transmissions. Practically, this indicates that allocating more power to the common signal ($w_{c}$) combined with higher friendly jamming power (${\bar{\rho }}_{J}$) effectively masks covert transmissions, providing system designers a flexible tradeoff between covertness and private data reliability.

**Fig 8:**
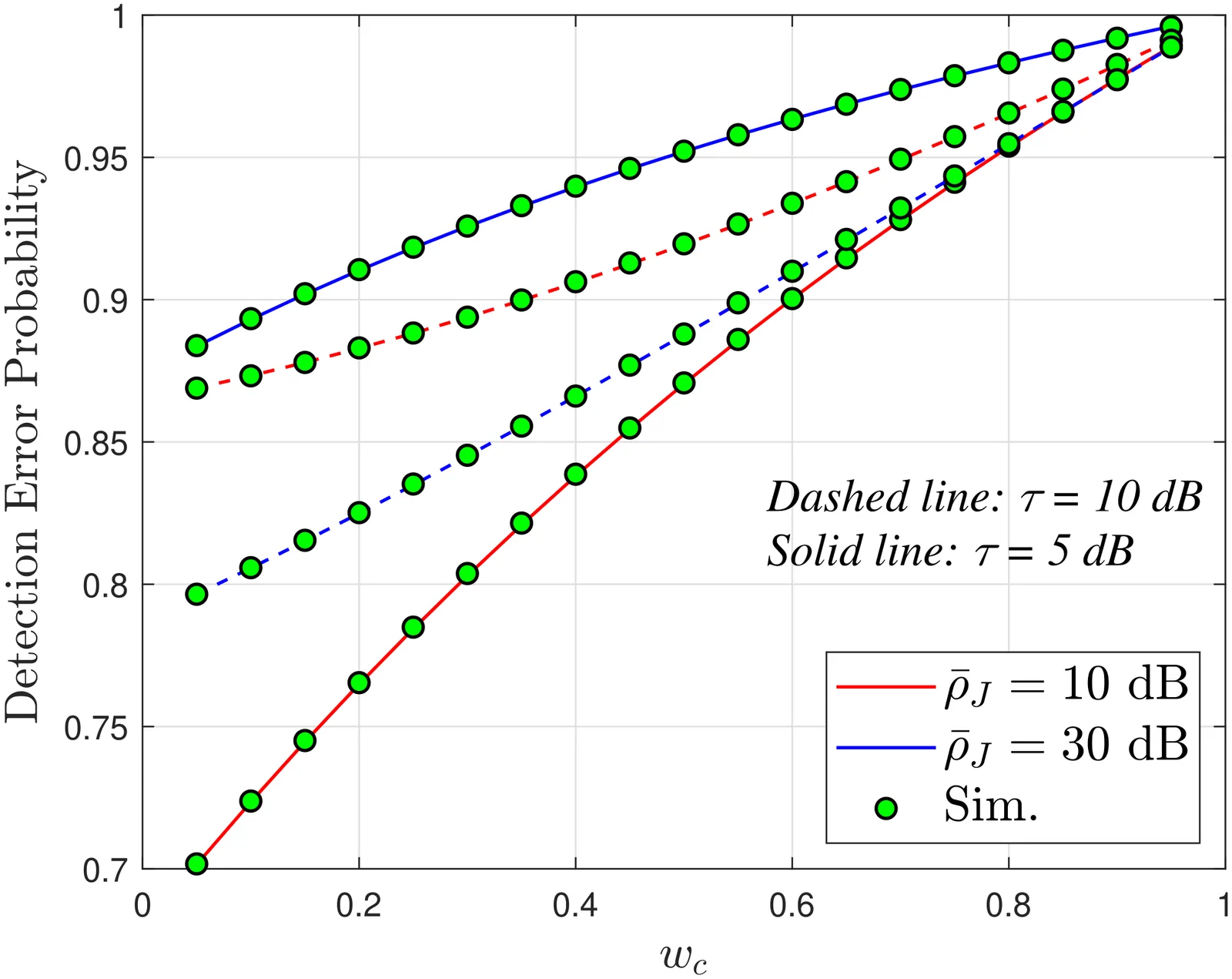
The DEP versus $w_{c}$, with *m* = 2, ℓ=0.5 and $\rho_{b}=30dB$.

Fig 9 presents the DEP versus the UAV altitude, denoted by $z_{U}$. Interestingly, it can be observed that the DEP exhibits a non-monotonic behavior with respect to the UAV height. Specifically, as $z_{U}$ increases from a low altitude, the DEP initially decreases until it reaches a minimum point, after which it increases again. This trend implies the existence of a specific UAV altitude that minimizes the detection error at the eavesdropper, representing the worst-case scenario for communication covertness. When the UAV is positioned at an extremely high altitude, the severe path loss dominates the channel conditions, thereby weakening the intercepted signal strength at $V_{E}$ and consequently driving the DEP back up. Furthermore, it is evident that different fading parameters (*m*) and detection thresholds (τ) significantly influence the covertness performance, shifting both the minimum DEP values and the optimal altitudes. In practice, network operators must avoid placing the UAV-BS near the critical altitude region (around 120–180 m) where DEP reaches its minimum, ensuring optimal 3D positioning to preserve high covertness.

**Fig 9:**
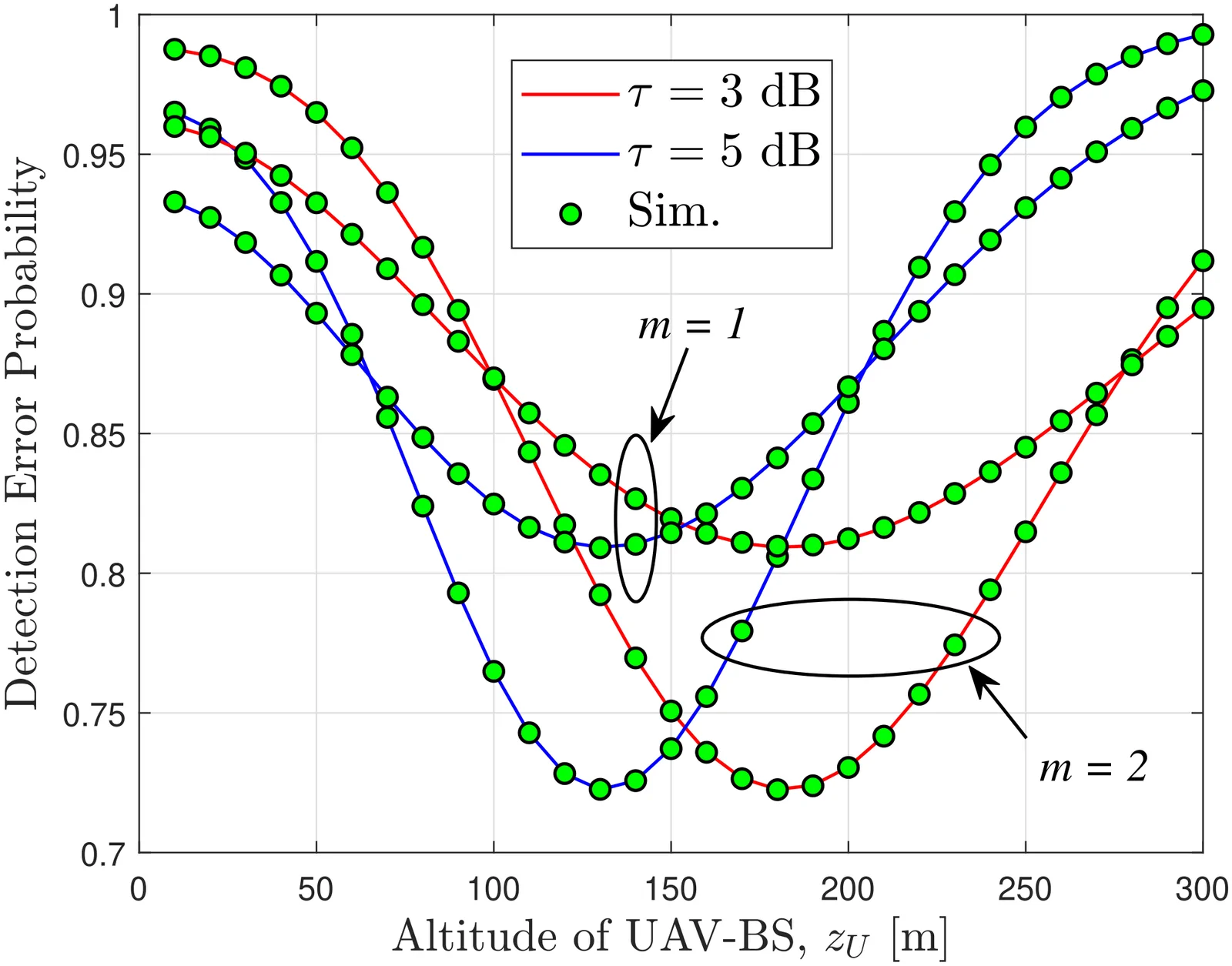
The DEP versus $z_{U}$, with *m* = 1,2, $w_{c}=0.2$, ${\bar{\rho }}_{J}=10dB$, ℓ=0.5 and $\rho_{b}=30dB$.

Finally, Fig 10 illustrates the IP at $V_{E}$ for decoding ${\bar{x}}_{S}$ versus $\rho_{b}$ under different Nakagami-*m* fading parameters. It is observed that the IP generally increases as $\rho_{b}$ grows, since higher transmit SNR improves the received signal quality at the monitoring node. Moreover, larger values of *m* tend to provide more stable channel conditions, which results in higher interception capability at $V_{E}$. This trend suggests a trade-off between transmission reliability and covert performance when the channel becomes less severe.

**Fig 10:**
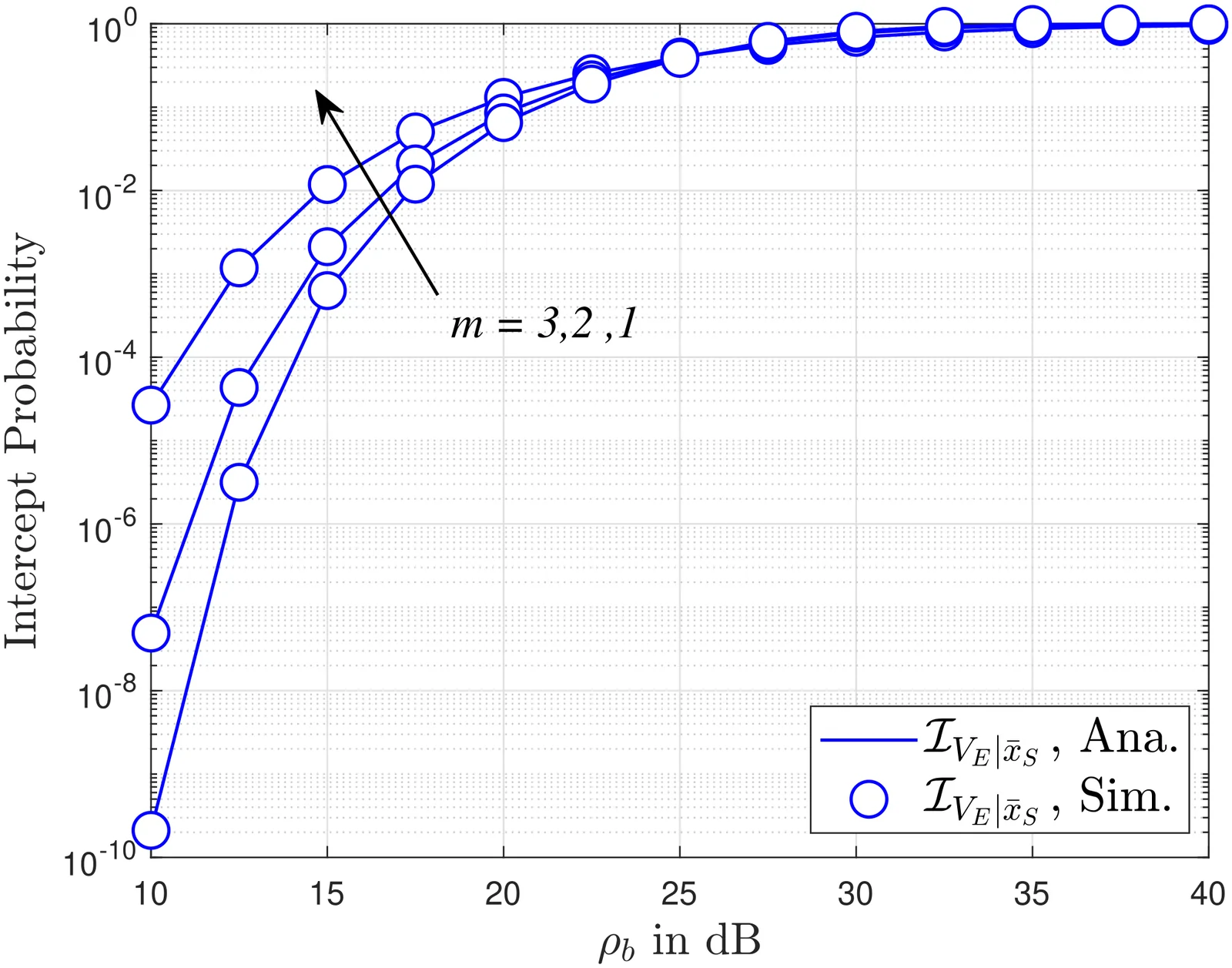
The IP versus $\rho_{b}$, with *m* = 1,2,3, $w_{c}=0.5$, ${\bar{\rho }}_{J}=30dB$ and η=0.5.

## 5 Conclusion

This paper investigated physical-layer covert communication in a UAV-assisted intelligent transportation system employing RSMA over Nakagami-*m* fading channels. In the considered framework, the UAV simultaneously transmits common and private information to multiple vehicular users, while a suspicious monitoring vehicle attempts to detect and intercept the covert transmissions. To enhance communication covertness, a friendly vehicular jammer with randomly varying transmit power was incorporated to increase the uncertainty at the monitoring node. Closed-form expressions for the OPs of legitimate vehicles and the IPs at the monitoring vehicle were derived to evaluate communication reliability and interception probability. In addition, an analytical approximation for the DEP was developed to quantify the system’s covertness performance. High-SNR asymptotic expressions were further developed to provide diversity insights into the proposed system. The analytical results were validated through extensive Monte Carlo simulations. The numerical results demonstrated that RSMA significantly improves transmission reliability while effectively degrading the monitoring and interception performance of the eavesdropper. Moreover, system parameters such as power allocation, fading severity, and jamming uncertainty were shown to play crucial roles in balancing reliability and covert communication performance. Future work may consider extending the proposed framework to multi-UAV cooperative transportation networks, adaptive jamming strategies, and practical mobility-aware vehicular channel models.

## Supporting information

S1 Appendix Proofs of Propositions 1–3. Appendix A: Proof of Proposition 1. Appendix B: Proof of Proposition 2. Appendix C: Proof of Proposition 3.
(PDF)
