# Supplementary material for: Physical layer covert communication for UAV-aided transportation systems via rate-splitting multiple access over Nakagami-m fading
Source: PLoS One. 2026 Nov 12;21(11):e0361873. doi: 10.1371/journal.pone.0361873 (PMC13759125; doi:10.1371/journal.pone.0361873)
Supplement: Supplementary file 1 [file pone.0361873.s001.pdf]

S1 Appendix. Proofs of Propositions 1–3

Supporting information for: *Physical Layer Covert Communication for UAV-Aided Transportation Systems via Rate-Splitting Multiple Access over Nakagami-m Fading.*
Equation numbers continue from the main manuscript; equations numbered (1)–(38) refer to the main manuscript.

Appendix A: Proof of Proposition 1

Substituting (9) into (21), the OP at V_S is given by

$$\begin{aligned}\mathcal{O}_{V_S} &= 1 - \Pr\left(|h_{US}|^2 > \frac{\varphi_{c,US}}{\rho_b}\left(\eta d_{JS}^{-\alpha}\rho_J |h_{JS}|^2 + 1\right)\right) \\ &= 1 - \int_0^{\bar{\rho}_J}\int_0^{\infty} f_{\rho_J}(x) f_{|h_{JS}|^2}(y) \\ &\quad \times \left[1 - F_{|h_{US}|^2}\left(\frac{\varphi_{c,US}}{\rho_b}\left(\eta d_{JS}^{-\alpha} xy + 1\right)\right)\right] dy\, dx,\end{aligned} \tag{39}$$

where $\phi_1 = w_c - \varepsilon_{th,S}^c (w_S + w_I)$ and $\varphi_{c,US} = \frac{\varepsilon_{th,S}^c}{\chi_{US}\phi_1}$. For notational simplicity, we assume identical noise variances at all receivers, i.e., $\sigma_S^2 = \sigma_I^2 = \sigma_E^2$. We set $\rho_b = P_b/\sigma_S^2 = P_b/\sigma_I^2 = P_b/\sigma_E^2$ to denote the average transmit signal-to-noise ratio (SNR), $\rho_J = P_J/\sigma_S^2$ to denote the instantaneous jammer SNR, and $\bar{\rho}_J = \bar{P}_J/\sigma_S^2$ to represent the maximum average jammer SNR.

By substituting (5), (6b), and (7a) into (39), and after some straightforward algebraic manipulations, $\mathcal{O}_{V_S}$ can be rewritten as

$$\begin{aligned}\mathcal{O}_{V_S} = 1 - &\frac{e^{-\mathcal{G}_{c,US}}\mu_{JS}^{m_{JS}}}{\bar{\rho}_J \Gamma(m_{JS})}\sum_{t=0}^{m_{US}-1}\frac{\mathcal{G}_{c,US}^t}{t!}\int_0^{\bar{\rho}_J}\int_0^{\infty} y^{m_{JS}-1} \\ &\times e^{-y\left(\mathcal{G}_{c,US}\eta d_{JS}^{-\alpha}x + \mu_{JS}\right)}\left(\eta d_{JS}^{-\alpha} xy + 1\right)^t dy\, dx,\end{aligned} \tag{40}$$

where $\mathcal{G}_{c,US} = \frac{\mu_{US}\varphi_{c,US}}{\rho_b}$. Using the binomial expansion $(x+a)^k = \sum_{v=0}^{k}\binom{k}{v}x^v a^{k-v}$, the OP in (40) becomes

$$\begin{aligned}\mathcal{O}_{V_S} = 1 - &\frac{e^{-\mathcal{G}_{c,US}}\mu_{JS}^{m_{JS}}}{\bar{\rho}_J \Gamma(m_{JS})}\sum_{t=0}^{m_{US}-1}\sum_{n=0}^{t}\binom{t}{n}\frac{\eta^n \mathcal{G}_{c,US}^t}{t! d_{JS}^{n\alpha}} \\ &\times \int_0^{\bar{\rho}_J} x^n \int_0^{\infty} y^{m_{JS}+n-1} e^{-y\left(\mathcal{G}_{c,US}\eta d_{JS}^{-\alpha}x + \mu_{JS}\right)} dy\, dx.\end{aligned} \tag{41}$$

By using [40, Eq. (3.351.3)], (41) reduces to

$$\begin{aligned}\mathcal{O}_{V_S} = 1 - &\frac{e^{-\mathcal{G}_{c,US}}\mu_{JS}^{m_{JS}}}{\bar{\rho}_J \Gamma(m_{JS})}\sum_{t=0}^{m_{US}-1}\sum_{n=0}^{t}\binom{t}{n}\frac{\eta^n \mathcal{G}_{c,US}^t}{t! d_{JS}^{n\alpha}} \\ &\times \Gamma(m_{JS}+n)\int_0^{\bar{\rho}_J}\frac{x^n}{\left(\mathcal{G}_{c,US}\eta d_{JS}^{-\alpha}x + \mu_{JS}\right)^{m_{JS}+n}} dx.\end{aligned} \tag{42}$$

Case 1: $m_{JS} \neq 1$. Let $r = \mathcal{G}_{c,US}\eta d_{JS}^{-\alpha}x + \mu_{JS}$. Then, $x = \frac{r-\mu_{JS}}{\mathcal{G}_{c,US}\eta d_{JS}^{-\alpha}}$ and $dx = \frac{dr}{\mathcal{G}_{c,US}\eta d_{JS}^{-\alpha}}$. Hence, (42) becomes

$$\begin{aligned}\mathcal{O}_{V_S} = 1 - &\frac{e^{-\mathcal{G}_{c,US}}\mu_{JS}^{m_{JS}}}{\bar{\rho}_J \Gamma(m_{JS})}\sum_{t=0}^{m_{US}-1}\sum_{n=0}^{t}\binom{t}{n}\frac{\mathcal{G}_{c,US}^{t-n-1}\Gamma(m_{JS}+n)}{t! \eta d_{JS}^{-\alpha}} \\ &\times \int_{\mu_{JS}}^{\mathcal{G}_{c,US}\eta d_{JS}^{-\alpha}\bar{\rho}_J + \mu_{JS}}\frac{(r-\mu_{JS})^n}{r^{m_{JS}+n}} dr.\end{aligned} \tag{43}$$

Expanding $(r-\mu_{JS})^n$ using the binomial theorem yields

$$\begin{aligned}\mathcal{O}_{V_S} = 1 - \frac{e^{-\mathcal{G}_{c,US}}}{\bar{\rho}_J \Gamma(m_{JS})} \sum_{t=0}^{m_{US}-1} \sum_{n=0}^{t} \sum_{l=0}^{n} \binom{t}{n} \binom{n}{l} \frac{(-1)^{n-l}}{t! \eta d_{JS}^{-\alpha}} \\ \times \mathcal{G}_{c,US}^{t-n-1} \mu_{JS}^{m_{JS}+n-l} \Gamma(m_{JS}+n) \\ \times \int_{\mu_{JS}}^{\mathcal{G}_{c,US}\eta d_{JS}^{-\alpha}\bar{\rho}_J+\mu_{JS}} r^{l-m_{JS}-n} dr.\end{aligned} \tag{44}$$

Evaluating the integral in (44) leads to

$$\begin{aligned}\mathcal{O}_{V_S} = 1 - \frac{e^{-\mathcal{G}_{c,US}}}{\bar{\rho}_J \Gamma(m_{JS})} \sum_{t=0}^{m_{US}-1} \sum_{n=0}^{t} \sum_{l=0}^{n} \binom{t}{n} \binom{n}{l} \frac{(-1)^{n-l}}{t! \eta d_{JS}^{-\alpha}} \\ \times \mathcal{G}_{c,US}^{t-n-1} \mu_{JS}^{m_{JS}+n-l} \Gamma(m_{JS}+n) \\ \times \left[\frac{\left(\mathcal{G}_{c,US}\eta d_{JS}^{-\alpha}\bar{\rho}_J+\mu_{JS}\right)^{l-m_{JS}-n+1} - \mu_{JS}^{l-m_{JS}-n+1}}{l-m_{JS}-n+1} \right].\end{aligned} \tag{45}$$

Case 2: $m_{JS}=1$. Here, $\mathcal{O}_{V_S}$ can be calculated by

$$\begin{aligned}\mathcal{O}_{V_S} = 1 - \frac{e^{-\mathcal{G}_{c,US}} \mu_{JS}}{\bar{\rho}_J} \sum_{t=0}^{m_{US}-1} \sum_{n=0}^{t} \binom{t}{n} \frac{\eta^n \mathcal{G}_{c,US}^{t} \Gamma(n+1)}{t! d_{JS}^{n\alpha}} \\ \times \int_0^{\bar{\rho}_J} \frac{x^n}{\left(\mathcal{G}_{c,US}\eta d_{JS}^{-\alpha} x + \mu_{JS}\right)^{n+1}} dx.\end{aligned} \tag{46}$$

By changing the variable to $r = \mathcal{G}_{c,US}\eta d_{JS}^{-\alpha}x + \mu_{JS}$, (46) transforms into

$$\begin{aligned}\mathcal{O}_{V_S} = 1 - \frac{e^{-\mathcal{G}_{c,US}} \mu_{JS}}{\bar{\rho}_J} \sum_{t=0}^{m_{US}-1} \sum_{n=0}^{t} \binom{t}{n} \frac{\mathcal{G}_{c,US}^{t-n-1} \Gamma(n+1)}{t! \eta d_{JS}^{-\alpha}} \\ \times \int_{\mu_{JS}}^{\mathcal{G}_{c,US}\eta d_{JS}^{-\alpha}\bar{\rho}_J+\mu_{JS}} \frac{(r-\mu_{JS})^n}{r^{n+1}} dr.\end{aligned} \tag{47}$$

Using the binomial theorem to expand $(r-\mu_{JS})^n$ as

$$(r-\mu_{JS})^n = \sum_{l=0}^{n} \binom{n}{l} (-1)^{n-l} \mu_{JS}^{n-l} r^l, \tag{48}$$

and substituting this into (47), the outage probability $\mathcal{O}_{V_S}$ is rewritten as

$$\begin{aligned}\mathcal{O}_{V_S} = 1 - \frac{e^{-\mathcal{G}_{c,US}}}{\bar{\rho}_J} \sum_{t=0}^{m_{US}-1} \sum_{n=0}^{t} \sum_{l=0}^{n} \binom{t}{n} \binom{n}{l} \frac{(-1)^{n-l}}{t! \eta d_{JS}^{-\alpha}} \\ \times \mu_{JS}^{n+1-l} \mathcal{G}_{c,US}^{t-n-1} \Gamma(n+1) \int_{\mu_{JS}}^{\mathcal{G}_{c,US}\eta d_{JS}^{-\alpha}\bar{\rho}_J+\mu_{JS}} r^{l-n-1} dr.\end{aligned} \tag{49}$$

Next, the integral term in (49) can be evaluated depending on the relation between l and n:

$$\int r^{l-n-1} dr = \begin{cases} \frac{r^{l-n}}{l-n}, & l \neq n, \\ \ln(r), & l = n. \end{cases} \tag{50}$$

Applying (50) to (49) and evaluating the definite integral over the interval $[\mu_{JS}, \mathcal{G}_{c,US}\eta d_{JS}^{-\alpha}\bar{\rho}_J + \mu_{JS}]$, the outage probability is obtained as

$$\mathcal{O}_{V_S} = 1 - \frac{e^{-\mathcal{G}_{c,US}}}{\bar{\rho}_J} \sum_{t=0}^{m_{US}-1} \sum_{n=0}^{t} \sum_{l=0}^{n} \binom{t}{n} \binom{n}{l} \frac{(-1)^{n-l}}{t! \eta d_{JS}^{-\alpha}} \times \mathcal{G}_{c,US}^{t-n-1} \mu_{JS}^{n+1-l} \Gamma(n+1) \Phi_{n,l}^{(1)}, \tag{51}$$

where $\Phi_{n,l}^{(1)}$ denotes the closed-form result of the definite integral defined in (22).

Combining the results obtained for the cases $m_{JS} \neq 1$ in (45) and $m_{JS} = 1$ in (51), the unified expression in (22) is obtained, which completes the proof.

Appendix B: Proof of Proposition 2

When $\rho_b \to \infty$, it follows from (22) that

$$\mathcal{G}_{c,US} \to 0. \tag{52}$$

Using the first-order Taylor expansion of the exponential function, we have

$$e^{-\mathcal{G}_{c,US}} \approx 1 - \mathcal{G}_{c,US}. \tag{53}$$

From (22), the dominant outage behavior is governed by the smallest power of $\mathcal{G}_{c,US}$. The summation term contains $\mathcal{G}_{c,US}^{t-n-1}$, where $0 \leq n \leq t \leq m_{US} - 1$. The lowest exponent occurs when

$$t = m_{US} - 1, \quad n = t. \tag{54}$$

Therefore, the leading term of the outage probability is proportional to

$$\mathcal{G}_{c,US}^{m_{US}} = \left(\frac{\mu_{US} \varphi_{c,US}}{\rho_b} \right)^{m_{US}}. \tag{55}$$

From Appendix A, the outage expression involves the following integral term:

$$I_k = \int_0^{\bar{\rho}_J} \frac{x^k}{(\mu_{JS} + Ax)^{m_{JS}+k}} dx, \tag{56}$$

where $A = \mathcal{G}_{c,US}\eta d_{JS}^{-\alpha}$.

At high SNR, $\mathcal{G}_{c,US} \to 0$, which implies $A \to 0$. Thus, the denominator in (56) can be expanded as

$$(\mu_{JS} + Ax)^{-(m_{JS}+k)} = \mu_{JS}^{-(m_{JS}+k)} \left(1 + \frac{Ax}{\mu_{JS}} \right)^{-(m_{JS}+k)}. \tag{57}$$

Applying the binomial expansion yields

$$\left(1 + \frac{Ax}{\mu_{JS}} \right)^{-(m_{JS}+k)} \approx 1 + \mathcal{O}(A). \tag{58}$$

Substituting (58) into (56) and retaining the dominant term, we obtain

$$I_k \approx \frac{1}{\mu_{JS}^{m_{JS}+k}} \int_0^{\bar{\rho}_J} x^k dx. \tag{59}$$

The integral in (59) can be computed as

$$\int_0^{\bar{\rho}_J} x^k dx = \frac{\bar{\rho}_J^{k+1}}{k+1}. \tag{60}$$

Therefore, the high-SNR approximation of I_k is expressed as

$$I_k \approx \frac{\bar{\rho}_J^{k+1}}{(k+1)\mu_{JS}^{m_{JS}+k}}. \tag{61}$$

Substituting (55) and (61) into (22), and rearranging the terms, the asymptotic outage probability is obtained as

$$\mathcal{O}_{V_S}^{\infty} = \frac{(\mu_{US}\varphi_{c,US})^{m_{US}}}{m_{US}!\,\rho_b^{m_{US}}} \sum_{k=0}^{m_{US}} \binom{m_{US}}{k} \frac{\eta^k d_{JS}^{-k\alpha} \bar{\rho}_J^k \Gamma(m_{JS}+k)}{(k+1)\Gamma(m_{JS})\mu_{JS}^k}. \tag{62}$$

This completes the proof.

Appendix C: Proof of Proposition 3

Based on (17) and (18), the false alarm probability p_f is derived as

$$\begin{aligned} p_f &= \Pr\left(D_1 \mid \mathcal{H}_0\right) \\ &= \Pr\left(\varsigma_0\rho_b|h_{UE}|^2 + d_{JE}^{-\alpha}\rho_J|h_{JE}|^2 > \Lambda\right), \end{aligned} \tag{63}$$

where $\Lambda = \frac{\tau}{\sigma_E^2\ell} - 1$. The probability can be further rewritten as

$$p_f = \Pr\left(|h_{UE}|^2 > \frac{\Lambda - d_{JE}^{-\alpha}\rho_J|h_{JE}|^2}{\varsigma_0\rho_b}\right). \tag{64}$$

To evaluate p_f, we must consider the CDF of the Nakagami-m fading channels and the uniform distribution of the jammer power. Let $X = \rho_J$ be a uniform random variable in $[0, \bar{\rho}_J]$ and $Y = |h_{JE}|^2$ follow a Gamma distribution. The total probability is calculated by conditioning on X and Y as follows:

$$p_f = \int_0^{\bar{\rho}_J}\int_0^{\infty} \Pr\left(|h_{UE}|^2 > \frac{\Lambda - d_{JE}^{-\alpha}xy}{\varsigma_0\rho_b}\right) f_Y(y) f_X(x) dy\, dx. \tag{65}$$

Due to the nature of the threshold, we partition the inner integral into two regions: Region 1 where the effective threshold is positive $\left(y < \frac{\Lambda}{d_{JE}^{-\alpha}x}\right)$, and Region 2 where the jamming power alone exceeds the threshold $\left(y \geq \frac{\Lambda}{d_{JE}^{-\alpha}x}\right)$, leading to a conditional detection probability of unity. Thus:

$$p_f = \frac{1}{\bar{\rho}_J}\int_0^{\bar{\rho}_J}\left[\int_0^{\frac{\Lambda}{d_{JE}^{-\alpha}x}} \bar{F}_{|h_{UE}|^2}\left(\frac{\Lambda - d_{JE}^{-\alpha}xy}{\varsigma_0\rho_b}\right) f_Y(y)dy + \int_{\frac{\Lambda}{d_{JE}^{-\alpha}x}}^{\infty} f_Y(y)dy\right] dx, \tag{66}$$

where $\bar{F}_{|h_{UE}|^2}(\cdot)$ is the complementary CDF (CCDF) of the Nakagami-m fading channel. Substituting the PDF of Y and the CCDF of $|h_{UE}|^2$, and applying the binomial expansion $(\Lambda - d_{JE}^{-\alpha}xy)^t = \sum_{l=0}^{t}\binom{t}{l}\Lambda^{t-l}(-1)^l(d_{JE}^{-\alpha}x)^l y^l$, the inner integral for Region 1 can be solved as

$$\begin{aligned} \mathcal{I}_1(x) &= \frac{\mu_{JE}^{m_{JE}} e^{-\frac{\Lambda\mu_{UE}}{\varsigma_0\rho_b}}}{\Gamma(m_{JE})} \sum_{t=0}^{m_{UE}-1} \frac{\mu_{UE}^t}{t!} \int_0^{\frac{\Lambda}{d_{JE}^{-\alpha}x}} y^{m_{JE}-1} \\ &\quad \times e^{-\mathcal{G}(x)y}\left(\frac{\Lambda - d_{JE}^{-\alpha}xy}{\varsigma_0\rho_b}\right)^t dy \\ &= \frac{\mu_{JE}^{m_{JE}} e^{-\frac{\Lambda\mu_{UE}}{\varsigma_0\rho_b}}}{\Gamma(m_{JE})} \sum_{t=0}^{m_{UE}-1}\sum_{l=0}^{t}\binom{t}{l} \frac{(-1)^l\Lambda^{t-l}\mu_{UE}^t x^l}{t!\varsigma_0^t\rho_b^t d_{JE}^{l\alpha}} \\ &\quad \times \int_0^{\frac{\Lambda}{d_{JE}^{-\alpha}x}} y^{m_{JE}+l-1} e^{-\mathcal{G}(x)y} dy, \end{aligned} \tag{67}$$

where $\mathcal{G}(x) = \mu_{JE} - \frac{\mu_{UE} d_{JE}^{-\alpha} x}{\varsigma_0 \rho_b}$. Making use of [40, Eq. (3.381.1)], $\mathcal{I}_1(x)$ is given by

$$\begin{aligned}\mathcal{I}_1(x) = \frac{\mu_{JE}^{m_{JE}} e^{-\frac{\Lambda \mu_{UE}}{\varsigma_0 \rho_b}}}{\Gamma(m_{JE})} \sum_{t=0}^{m_{UE}-1} \sum_{l=0}^{t} \binom{t}{l} \frac{(-1)^l \Lambda^{t-l}}{t! \varsigma_0^t \rho_b^t d_{JE}^{l\alpha}} \\ \times \frac{\mu_{UE}^t x^l}{\mathcal{G}(x)^{m_{JE}+l}} \gamma\left(m_{JE}+l, \frac{\mathcal{G}(x)\Lambda}{d_{JE}^{-\alpha} x}\right).\end{aligned} \tag{68}$$

Next, we evaluate the second integral term, $\mathcal{I}_2(x)$, which corresponds to the strong jamming regime. In Region 2, the interference power from the jammer is sufficiently large such that $d_{JE}^{-\alpha} P_J |h_{JE}|^2 \geq \Lambda$. Consequently, the threshold condition inside the probability function becomes non-positive. Since the channel gain $|h_{UE}|^2$ is strictly non-negative, this condition is satisfied with probability 1. Thus, $\mathcal{I}_2(x)$ simplifies to the probability that the jamming channel gain Y exceeds the critical value $Y_{\text{lim}} = \frac{\Lambda}{d_{JE}^{-\alpha} x}$:

$$\mathcal{I}_2(x) = \int_{Y_{\text{lim}}}^{\infty} 1 \cdot f_Y(y) dy = \int_{Y_{\text{lim}}}^{\infty} \frac{\mu_{JE}^{m_{JE}}}{\Gamma(m_{JE})} y^{m_{JE}-1} e^{-\mu_{JE} y} dy. \tag{69}$$

To solve this integral, we employ the change of variable $t = \mu_{JE} y$, which yields $dy = \frac{1}{\mu_{JE}} dt$. The lower limit of integration changes to $\mu_{JE} Y_{\text{lim}}$. Substituting these into the integral gives

$$\mathcal{I}_2(x) = \frac{1}{\Gamma(m_{JE})} \int_{\mu_{JE} Y_{\text{lim}}}^{\infty} t^{m_{JE}-1} e^{-t} dt. \tag{70}$$

By definition, the integral term on the right-hand side corresponds to the upper incomplete gamma function, denoted as $\Gamma(s, z) = \int_z^{\infty} t^{s-1} e^{-t} dt$ [40, Eq. (3.351.2)]. Therefore, the closed-form expression for the second region is derived as

$$\mathcal{I}_2(x) = \frac{\Gamma\left(m_{JE}, \frac{\mu_{JE}\Lambda}{d_{JE}^{-\alpha} x}\right)}{\Gamma(m_{JE})}. \tag{71}$$

Combining the results from $\mathcal{I}_1(x)$ and $\mathcal{I}_2(x)$ into the Gauss-Chebyshev summation yields the final analytical expression for the false alarm probability presented in (35).

This completes the proof.

References

[40] Gradshteyn IS, Ryzhik IM. Table of Integrals, Series, and Products. 7th ed. Academic Press; 2007. (Reference numbering follows the main manuscript.)
